# The F-box protein FKF1 inhibits dimerization of COP1 in the control of photoperiodic flowering

**DOI:** 10.1038/s41467-017-02476-2

**Published:** 2017-12-22

**Authors:** Byoung-Doo Lee, Mi Ri Kim, Min-Young Kang, Joon-Yung Cha, Su-Hyun Han, Ganesh M. Nawkar, Yasuhito Sakuraba, Sang Yeol Lee, Takato Imaizumi, C. Robertson McClung, Woe-Yeon Kim, Nam-Chon Paek

**Affiliations:** 1 Department of Plant Science, Plant Genomics and Breeding Institute, Research Institute of Agriculture and Life Sciences, Seoul National University, Seoul, 08826 Republic of Korea; 2 Division of Applied Life Science (BK21Plus), PMBBRC & IALS, Gyeongsang National University, Jinju, 52828 Republic of Korea; 3 Department of Biology, University of Washington, Seattle, WA 98195-1800 USA; 4 Department of Biological Sciences, Dartmouth College, Hanover, NH 03755-3563 USA

## Abstract

In *Arabidopsis thaliana*, CONSTANS (CO) plays an essential role in the regulation of photoperiodic flowering under long-day conditions. CO protein is stable only in the afternoon of long days, when it induces the expression of *FLOWERING LOCUS T* (*FT*), which promotes flowering. The blue-light photoreceptor FLAVIN-BINDING, KELCH REPEAT, F-BOX1 (FKF1) interacts with CO and stabilizes it by an unknown mechanism. Here, we provide genetic and biochemical evidence that FKF1 inhibits CONSTITUTIVE PHOTOMORPHOGENIC1 (COP1)-dependent CO degradation. Light-activated FKF1 has no apparent effect on COP1 stability but can interact with and negatively regulate COP1. We show that FKF1 can inhibit COP1 homo-dimerization. Mutation of the coiled-coil domain in COP1, which prevents dimer formation, impairs COP1 function in coordinating flowering time. Based on these results, we propose a model whereby the light- and day length-dependent interaction between FKF1 and COP1 controls CO stability to regulate flowering time.

## Abstract (web summary)

CONSTANS promotes flowering under long-day conditions in *Arabidopsis* but is rapidly degraded in short-day conditions. Here the authors show that the blue-light photoreceptor FKF1 can interact with the E3 ligase COP1 in a light-dependent manner and prevent degradation of CO in long-day conditions.

## Introduction

Most flowering plants bloom in response to seasonal changes in environmental factors such as day length and temperature. In the model dicot plant *Arabidopsis thaliana*, flowering time is mainly regulated by the photoperiodic, autonomous, gibberellin, and vernalization pathways^1^. These signaling pathways converge to induce expression of the florigen gene *FLOWERING LOCUS T* (*FT*), which encodes a mobile protein that can induce the shoot apical meristem to make the transition from vegetative to reproductive development^2,3^. In the photoperiodic pathway, CONSTANS (CO) has a major role in inducing *FT* transcription, although other regulators also independently affect *FT* expression^4–6^. *CO* encodes a zinc finger-type transcription factor containing two B-boxes and a CO, CO-LIKE, and TOC1 (CCT) domain^7^. CO directly binds to the *FT* promoter and activates its transcription^8^. Levels of *CO* mRNA are regulated in a circadian manner: the *CO* mRNA is abundant during the daytime under long-day (LD) conditions and during the nighttime under short-day (SD) conditions^1,9^. However, *FT* transcription, controlled by CO, differs remarkably between LD and SD conditions because light signals tightly regulate CO at the posttranslational level. Elucidation of these regulatory mechanisms showed that FLAVIN-BINDING, KELCH REPEAT, F-BOX1 (FKF1) and CONSTITUTIVE PHOTOMORPHOGENIC1 (COP1) control CO stability^10–12^.

FKF1 is a key component of the SKP1/CUL1/F-box (SCF)-type E3 ligase complex and has three domains (LOV, F-box, and KELCH-repeat). FKF1 functions as a blue-light receptor in which the LOV domain binds to a flavin mononucleotide chromophore^13^. In photoperiodic flowering, FKF1 positively regulates CO at both the transcriptional and posttranslational levels^11,12^. In the morning, CYCLING DOF FACTOR (CDF) transcription factors (CDF1, CDF2, CDF3, and CDF5) repress the expression of *CO* and *FT*. In the afternoon of LD, *FKF1* is expressed and light-activated FKF1 interacts with GIGANTEA (GI) to degrade CDFs; this induces transcription of *CO* and *FT*, leading to early flowering. In SD, by contrast, *FKF1* is mainly expressed after dusk, and un-illuminated FKF1 has a decreased affinity for GI, resulting in the persistence of CDFs and thus a failure to induce *CO* transcription^11^. As recently reported, light-activated FKF1 also interacts with and stabilizes CO to activate *FT* transcription^12^.

In darkness, CO is ubiquitinated by COP1 in the nucleus and is degraded by 26S proteasome-dependent proteolysis^10,14,15^. Weak mutants of *COP1* (a strong *cop1* allele is lethal) exhibit very early flowering in SD and accumulate high levels of CO in darkness^15^. COP1 is a RING-type E3 ubiquitin ligase containing three domains (RING, coiled-coil (CC), and WD40-repeat)^16^. COP1 partitions between the nucleus and cytoplasm in a light-dependent manner^17^ and forms homodimers through the CC domain; the homo-dimerization is required for COP1 function and subcellular localization^18,19^. COP1 associates with SUPPRESSOR OF PHYTOCHROME A 1 (SPA1) to form a (COP1)_2_(SPA1)_2_ tetramer. Homo- and hetero-dimerization of COP1 have important roles in COP1 function^19,20^. The (COP1)_2_(SPA1)_2_ tetramer is involved in poly-ubiquitination and destabilization of CO in the dark^21^. In addition to CO, COP1 promotes destabilization of several nuclear proteins involved in flowering time and photomorphogenesis^21–23^.

Thus, the E3 ubiquitin ligases FKF1 and COP1 play critical roles in controlling photoperiodic flowering by directly regulating CO stability^10,12,15^, as FKF1 stabilizes CO in the light and COP1 destabilizes CO in the dark. Other important regulators also affect the stability of CO: (i) in the morning, HOS1 and phytochrome B (phyB) decrease CO stability^14,24,25^, (ii) in the afternoon of LD, phyA, cryptochrome 2 (cry2), and FKF1 increase CO stability^10,12,26,27^, and (iii) in darkness, COP1 mediates degradation of CO^15^.

Here, we provide evidence that FKF1 acts as an upstream negative regulator of COP1. FKF1 and COP1 regulate CO stability and photoperiodic flowering. FKF1 can interact with COP1 and reduce COP1 activity in a day-length-dependent manner. We suggest that posttranslational control of CO stability, mediated by negative regulation of COP1 by FKF1, promotes early flowering in LD.

## Results

### FKF1 negatively regulates COP1 in photoperiodic flowering

**Fig. 1:**
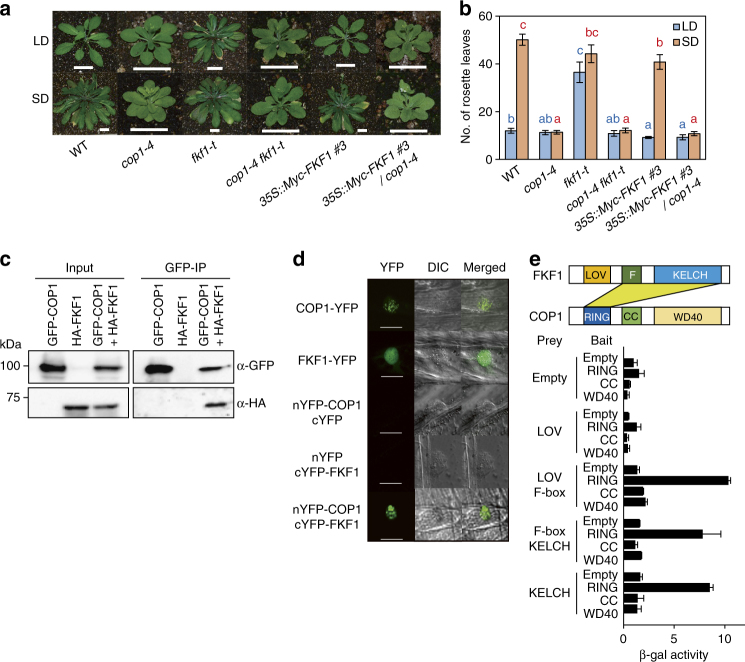
FKF1 genetically and physically interacts with COP1. **a** Phenotypes of plants containing *cop1-4* and/or *fkf1-t* mutations or overexpressing *Myc-FKF1* at bolting in LD (16-h light: 8-h dark) or SD (9-h light: 15-h dark). Scale bars, 2 cm. **b** *COP1* is epistatic to *FKF1* in the photoperiodic pathway of floral induction. Flowering time was measured by the number of rosette leaves at bolting. The blue and red letters indicate significant differences among genotypes in LD and SD, respectively (Duncan’s multiple range test). Data are means ± s.d. of 15 plants. **c** Co-IP results demonstrating the interaction of FKF1 with COP1 in the leaves of *N. benthamiana*. **d** BiFC visualization of the COP1–FKF1 interaction in the nucleus of an onion epidermal cell. For the COP1–FKF1 interaction, the two BiFC constructs (nYFP-COP1 and cYFP-FKF1) were transiently co-expressed in cell layers. Scale bars, 40 μm. **e** Both F-box and KELCH domains of FKF1 bind to the RING domain of COP1 in yeast two-hybrid assays. **e** Data are means ± s.d. of three replicates

To investigate whether COP1 and FKF1 act in the same genetic pathway of flowering-time regulation, we generated *cop1-4 fkf1-t* double mutants in which *cop1-4*, a weak mutant allele, carries a premature stop codon at the 283rd amino acid, and *fkf1-t* (SALK_059480) is a T-DNA insertion mutant^28,29^. Since *cop1-4 gi-1* mutants flowered as late as *gi-1* in both LD and SD^23^, we speculated that *cop1-4 fkf1-t* mutants would flower as late as *fkf1-t* in both photoperiods. However, *cop1-4 fkf1-t* flowered as early as *cop1-4* mutants in both LD and SD (Fig. 1a, b; Supplementary Table 1). Furthermore, we found that the *FKF1*-overexpressing plants, such as *35S::Myc-FKF1* #3 and *35S::FKF1* #18, showed an early-flowering phenotype compared with wild type (WT, Col-0), and *35S::Myc-FKF1* #3/*cop1-4* plants also flowered earlier than WT but similar to *cop1-4* in LD. In SD, both *35S::Myc-FKF1* #3 and *35S::FKF1* #18 flowered earlier than WT, and *35S::Myc-FKF1* #3/*cop1-4* flowered as early as *cop1-4*. These data indicate that *cop1* is epistatic to *fkf1*, and FKF1 inhibits COP1 mainly in LD. Considering these results, we concluded that FKF1 functions as an upstream negative regulator of COP1 in the floral induction pathway.

### FKF1 interacts with COP1 in vivo

The genetic analysis indicated that FKF1 and COP1 act in the same pathway of photoperiodic flowering. Since both COP1 and FKF1 function as E3-ubiquitin ligases in proteasome-mediated proteolysis of their target proteins^13,21^, we examined whether FKF1 directly regulates COP1. We first tested whether FKF1 and COP1 can physically interact and found that FKF1 interacts with COP1 in yeast two-hybrid assays, primarily via the RING domain of COP1 (Supplementary Fig. 1). This interaction between FKF1 and COP1 was also observed in planta by co-immunoprecipitation (Co-IP) following transient expression in *Nicotiana benthamiana*^30^ and by bimolecular fluorescence complementation (BiFC) assays in onion epidermal cells (Fig. 1c, d). Next, to map the interacting domains of FKF1 and COP1, we separated COP1 into three domains: (1) RING, (2) CC, and (3) WD40. Also, we separated FKF1 into four domains: (1) LOV, (2) LOV + F-box, (3) F-box + KELCH, and (4) KELCH domains. In yeast two-hybrid assays, we found that both the F-box and KELCH domains of FKF1 interacted with the RING domain of COP1 (Fig. 1e). The domain interaction between FKF1 and COP1 was further confirmed by BiFC (Supplementary Fig. 2) and Co-IP assays (Supplementary Fig. 3), consistent with the interaction results from yeast two-hybrid assays. Together with the genetic data, these findings suggest that FKF1 interacts with and negatively regulates COP1 function in flowering.

### CO is stabilized in the *cop1* mutant independently of FKF1

**Fig. 2:**
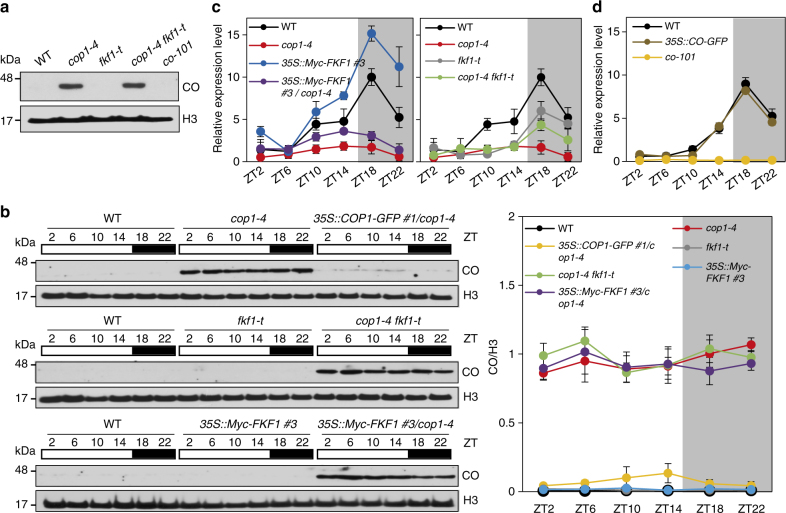
CO accumulates to high levels in the *cop1-4* background regardless of FKF1. **a** CO accumulation at ZT15 in 10-day-old seedlings under LD. **b** CO accumulation in various plants. Plants were grown for 10 days under LD and harvested every 4 h over the course of a day. Nuclear protein-enriched fractions were immunoblotted using an anti-CO antibody (α-CO) to measure CO levels and an anti-H3 antibody (α-H3) for a loading control. Data are means ± s.d. from at least three biological repeats. **c** Relative abundance of *CO* mRNA during a day in 10-day-old seedlings. **d** Native *CO* mRNA levels during a day in *35S::CO-GFP* transgenic plants. To detect the native *CO* mRNA, a specific primer was designed from the 3′-UTR region of the *CO* mRNA sequence. **c**, **d** For RT-qPCR, the relative expression level of each gene was normalized to the mRNA level of *ACTIN* (AT3G18780) as a loading control. Data are means ± s.d. from three biological replicates

Previous work reported that both FKF1 and COP1 E3-ubiquitin ligases interact with CO to control its function in LD-dependent early flowering antagonistically^12,15^. FKF1 and COP1 increase and decrease the stability of CO, respectively, because CO levels decrease in *fkf1-2* mutants and increase in *cop1-4* mutants. Thus, we tested whether the presence or absence of FKF1 activity affects CO stability in the *cop1-4* background (Fig. 2a, b). We first analyzed CO levels at ZT15 (1 h before darkness) when FKF1 expression and activity are the highest in LD. In *cop1-4 fkf1-t* mutants, CO accumulated to levels similar to those in *cop1-4*, but CO was not detected in either WT or the *fkf1-t* mutant (Fig. 2a). Next, we analyzed CO levels every 4 h during the course of a day in LD, and found that CO levels were not altered in the *cop1-4* background regardless of FKF1 genotype. CO levels were nearly constant during day and night in *cop1-4*, *cop1-4 fkf1-t*, and *35S::Myc-FKF1/cop1-4* plants, but CO was hardly detectable in WT, *fkf1-t*, or *35S::Myc-FKF1* #3 plants (Fig. 2b).

Finally, we analyzed the relative abundance of *CO* mRNA in different mutant backgrounds (Fig. 2c, d). *CO* mRNA levels decreased in *fkf1-t*, and increased in *35S::Myc-FKF1*, consistent with the observation that FKF1 functions to degrade CDF1, a negative regulator of *CO* transcription^11^. Interestingly, despite high accumulation of CO, the *CO* mRNA levels were lower in the *cop1* mutant background, including in *cop1-4*, *cop1-4 fkf1-t*, and *35S::Myc-FKF1 #3/cop1-4* (Fig. 2c). However, it did not appear that high accumulation of CO negatively affected *CO* transcription, because native *CO* mRNA levels in *35S::CO-GFP* were similar to those of WT (Fig. 2d). Taking these observations and those of a previous study^12^ together, we suggest that FKF1 increases CO stability by reducing COP1 function in the late afternoon of LD to induce flowering.

### FKF1 does not affect COP1 stability

**Fig. 3:**
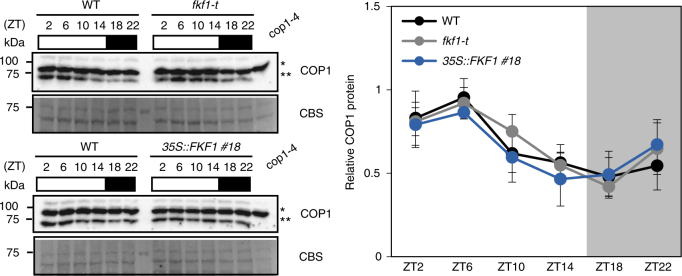
The FKF1–COP1 interaction does not affect COP1 stability. The diurnal patterns of COP1 levels are shown in wild type (Col-0), *fkf1-t*, and *35S::FKF1 #18* under LD. The 10-day-old seedlings were harvested every 4 h over the course of a day. Total protein (70 μg) extracted from each sample was immunoblotted to measure the COP1 levels using an anti-COP1 polyclonal antibody. The intensity of each COP1 band (**, lower band) was normalized to the non-specific band (*, upper band) in each lane. Quantitative COP1 data were exported from ImageJ (https://imagej.nih.gov/ij/index.html). Data are means ± s. d. from five immunoblot replicates

To examine whether FKF1 negatively regulates the stability of COP1, because FKF1 has E3-ubiquitin ligase activity^31^, we generated an anti-COP1 polyclonal antibody, and analyzed COP1 levels in *fkf1-t* and *35S::FKF1* #18 plants over the course of a day. Unexpectedly, we found that steady-state levels of COP1 persisted in both *fkf1-t* and *35S::FKF1 #18* compared with WT (Fig. 3; Supplementary Fig. 4), indicating that FKF1 does not destabilize COP1. Similarly, we found that FKF1 levels were not significantly altered in *cop1-4* or *35S::TAP-COP1* plants (Supplementary Fig. 5). These results indicate that the FKF1–COP1 interaction does not affect the stability of either protein.

### FKF1 can inhibit COP1 homo-dimerization

**Fig. 4:**
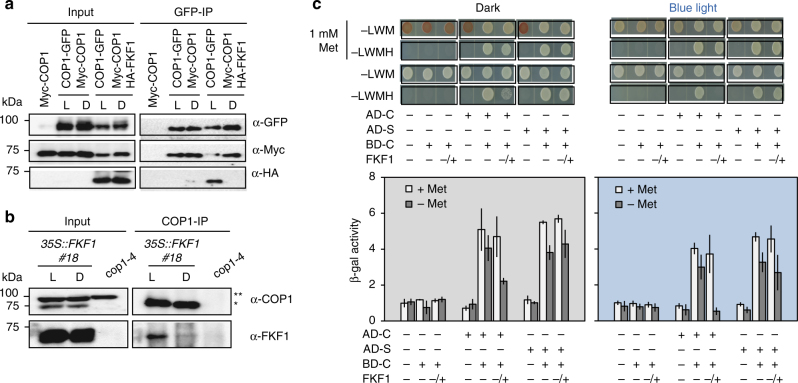
FKF1 inhibits COP1 homo-dimerization in a light-dependent manner. **a** FKF1 requires light to interact with COP1 and inhibit COP1 dimerization. In the absence of FKF1, COP1 dimerization occurs in both light and dark conditions. In the presence of FKF1, FKF1 interacts with COP1 only under light in *N. benthamiana*. Two days after co-infiltration, tobacco leaves were harvested under light (L) and dark (D) in LD. **b** Light-dependent interaction between FKF1 and COP1 in *35S:FKF1* #18. The 10-day-old seedlings were harvested in the light period (L) and dark period (D) in LD. * indicates COP1 and ** indicates non-specific bands derived from an anti-COP1 antibody. FKF1 was detected by an anti-FKF1 antibody. **c** FKF1 inhibits COP1 dimerization light-dependently in yeast three-hybrid assays. The interactions were evaluated based on yeast cell survival (upper panel), and were further confirmed by β-galactosidase (β-Gal) activity (lower panel). Data are means ± s.d. of three samples. Met, methionine; −LWM, the absence of Leu/Trp/Met in the yeast media; −LWMH, the absence of Leu/Trp/Met/His; AD, activation domain; BD, binding domain; C, COP1; S, SPA1. − or + represent the presence or absence of protein expression, respectively. In FKF1, −/+ indicates the absence of FKF1 in the first and second panels due to the addition of 1 mM Met and the presence of FKF1 in the third and fourth panels because 1 mM Met was not added to the yeast media

Since FKF1 does not affect the stability of COP1, we assumed that the FKF1–COP1 interaction decreases COP1 activity. COP1 interacts with SPA1 to form a (COP1)_2_(SPA1)_2_ tetramer, and homo- and hetero-dimerization of COP1 is important for its biological function^19,20^. Therefore, we speculated that the FKF1–COP1 interaction prevents COP1–COP1 dimerization, the COP1–SPA1 interaction, or both, thus decreasing COP1 activity and increasing CO stability in the late afternoon of LD. To test these possibilities, we performed Co-IP assays in *N. benthamiana*. We found that COP1 dimerization occurred under both light and dark conditions in the absence of FKF1. Surprisingly, *FKF1* overexpression diminished COP1 dimerization in the light but not in the dark (Fig. 4a). In the light, COP1 dimerization was severely decreased and, instead of forming homodimers, COP1 interacted with FKF1. In the dark, COP1 dimerization occurred normally and COP1 did not interact with FKF1. Next, we tested whether the in vivo interaction of FKF1 with COP1 depends on light. For this, we used Co-IP assays with *35S::FKF1 #18* plants, which revealed that the in vivo interaction requires light (Fig. 4b).

We further used yeast three-hybrid assays to test whether FKF1 inhibits COP1–COP1 homo-dimerization and/or COP1–SPA1 hetero-dimerization. In these assays, *FKF1* transcription was controlled by the Met-repressible *pMET25* promoter. We analyzed the inhibition of COP1 homo-dimerization by FKF1 under blue light or in darkness, and found that FKF1 inhibits COP1 homo-dimerization more under blue light than in darkness (Fig. 4c; Supplementary Fig. 6). COP1 homo-dimerization was completely inhibited by FKF1 under blue light and was reduced in methionine-deficient conditions in the dark, based on both yeast colony survival and β-galactosidase activity. Moreover, we found that FKF1 does not inhibit the COP1–SPA1 interaction, regardless of light conditions. These results strongly suggest that in yeast, FKF1-mediated inhibition of COP1 dimerization is promoted by blue light, although some of the activity remains in darkness. Taken together, these results suggest that in *Arabidopsis*, blue-light-activated FKF1 can interact with and attenuate COP1 homo-dimerization.

### FKF1 partially inhibits COP1 during hypocotyl elongation

The COP1 E3-ubiquitin ligase mediates degradation of specific target proteins, including HY5, HYH, LAF1, HFR1, BBX24/STO, BBX4/COL3, and BBX22/LZF1/STH3 transcription factors, all of which are involved in light signaling and photomorphogenesis in *Arabidopsis*^18,21^. Therefore, we further examined whether FKF1 also negatively affects COP1 function in hypocotyl elongation (Supplementary Fig. 7a; Supplementary Table 2). The hypocotyls of *fkf1-t* seedlings were as long as those of WT, regardless of day-length conditions (LD/SD/constant darkness; DD). Interestingly, hypocotyls of both *35S::FKF1 #18* and *35S::Myc-FKF1 #3* were significantly shorter than those of WT and *fkf1-t* in SD and slightly shorter in LD, but this was not statistically significant, and they were as long as WT in DD. These results suggest that *FKF1* overexpression negatively regulates COP1 in hypocotyl elongation only in SD.

Next, we analyzed HY5 levels in *35S::Myc-FKF1 #3* and *fkf1-t* plants (Supplementary Fig. 7b), since HY5 is one of major regulators of hypocotyl elongation, although other COP1 target proteins are also involved in this process^21^. HY5 stability in WT depends on the light period, as HY5 is more stable in LD than in SD, and not detected in DD. However, we could not find any evidence that HY5 becomes more stable in *35S::Myc-FKF1 #3* in SD. Thus, we concluded that FKF1 negatively affects COP1 function in hypocotyl growth in SD when *FKF1* is constitutively overexpressed, and this is seemingly not related to the regulation of HY5 stability.

### COP1 mutants that are unable to dimerize do not promote flowering

**Fig. 5:**
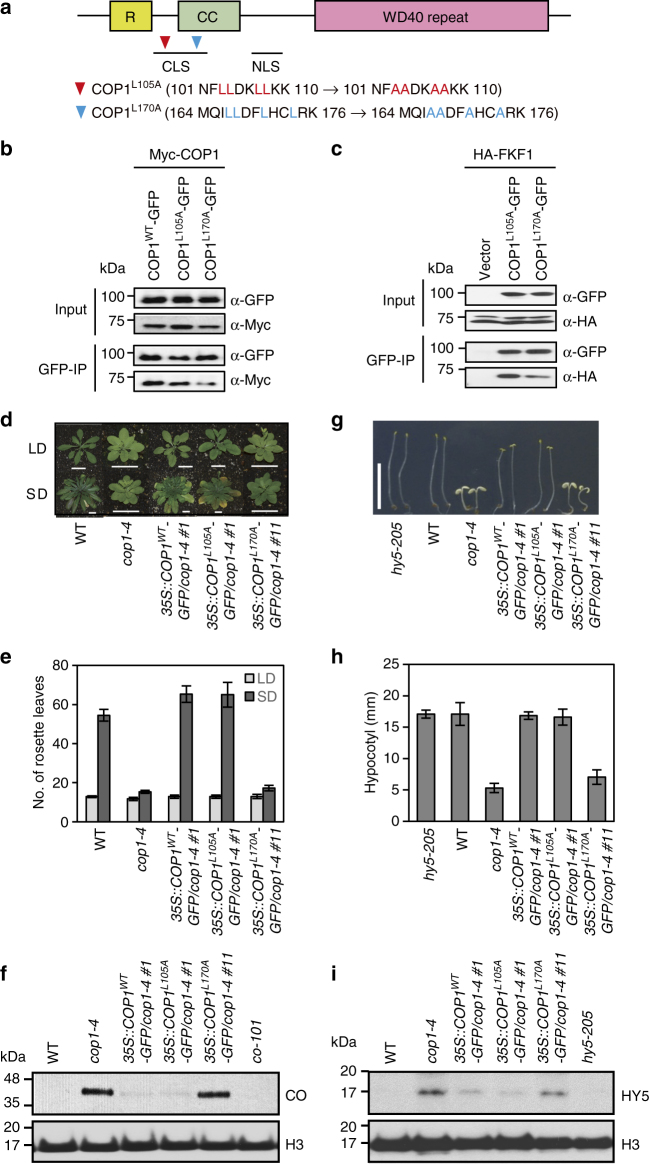
COP1 variants that are unable to homo-dimerize are non-functional. **a** Mutation sites in the CLS motif of COP1. **b** COP1^L170A^ forms dimers poorly when compared with COP1^WT^ or COP1^L105A^ in *N. benthamiana*. **c** FKF1 interacts more weakly with COP1^L170A^ (poor dimer formation) than COP1^L105A^ (normal dimer formation) in *N. benthamiana*. **d** Various transgenic plants at bolting under LD and SD. Scale bars, 2 cm. **e** Flowering times of the plants in **d**. The number of rosette leaves at bolting represents the flowering time of each genotype. Data are means ± s.d. of 20 plants. **f** CO accumulation of each genotype in **d**. Ten-day-old plants were harvested at ZT15 (1 h before dark) in LD. **g** Hypocotyl elongation of plants in **d** grown for 5 days in complete darkness. Scale bars, 1 cm. **h** Hypocotyl length of transgenic plants in darkness. Data are means ± s.d. of 20 plants. **i** HY5 accumulation in various transgenic plants

COP1 forms a homodimer and/or a heterodimer with SPA1 through the CC domain and finally forms a (COP1)_2_(SPA1)_2_ tetramer for its functional activity^18–20^. When COP1 dimerization is prevented, it is not functional in photomorphogenesis. To examine the effect of COP1 dimerization on flowering, we prepared mutated cDNAs using WT (Col-0) *COP1* (*COP1*^*WT*^), and the mutant versions *COP1*^*L105A*^ and *COP1*^*L170A*^ which were previously reported to undergo normal or poor dimer formation, respectively^19^ (Fig. 5a). First, we tested the binding between COP1 and mutated COP1, and FKF1 and mutated COP1 in *N. benthamiana*. The COP1^WT^–COP1^L105A^ Co-IP signal was nearly the same as that of COP1^WT^–COP1^WT^, while that of COP1^WT^–COP1^L170A^ was much weaker consistent with a prior publication^19^. This indicates that COP1 homo-dimerization requires the L170 residue of COP1 (Fig. 5b; Supplementary Fig. 8). Similarly, FKF1 also interacted with COP1^WT^ and COP1^L105A^ much more strongly than with COP1^L170A^ (Fig. 5c).

To examine the effect of these alterations on COP1 function in flowering time, we also generated transgenic plants carrying the *35S::COP1*^*WT*^*-GFP, 35S::COP1*^*L105A*^*-GFP*, and *35S::COP1*^*L170A*^*-GFP* constructs in the *cop1-4* background. Both *35S::COP1*^*WT*^*-GFP* and *35S::COP1*^*L105A*^*-GFP* transgenes were able to fully rescue the early-flowering phenotype of *cop1-4*, whereas *35S::COP1*^*L170A*^*-GFP* failed to delay flowering in SD (Fig. 5d, e; Supplementary Table 3). Moreover, the degree of COP1 dimerization was inversely proportional to CO levels in the late afternoon of LD. The plants carrying *35S::COP1*^*L170A*^*-GFP/cop1-4* had much higher CO levels than the *35S::COP1*^*WT*^*-GFP/cop1-4* and *35S::COP1*^*L105A*^*-GFP/cop1-4* plants (Fig. 5f). *35S::COP1*^*L170A*^*-GFP/cop1-4* plants flower early, similar to *cop1-4*. These results show that the L170A variant that is unable to dimerize not only is non-functional in regulation of seed color as previously reported^19^ but also in regulation of flowering time.

Finally, we examined whether the COP1 variants differed in their ability to destabilize HY5 for hypocotyl elongation in the dark (Fig. 5g, h; Supplementary Fig. 9, Supplementary Table 4). The COP1^WT^-GFP and COP1^L105A^-GFP fusion proteins, which form dimers normally, complemented the short-hypocotyl and cotyledon-expansion phenotypes of the *cop1-4* mutant. However, COP1^L170A^-GFP, which forms dimers poorly, did not rescue the defect. In *cop1-4* mutants, HY5 was almost completely degraded in darkness by either COP1^WT^-GFP or COP1^L105A^-GFP, but not by COP1^L170A^-GFP (Fig. 5i). Although we cannot rule out that these effects may be due to some other effect of the L170A mutation, these results are consistent with the level of COP1 dimerization correlating with the level of its functional activity in the timing of flowering and photomorphogenesis.

## Discussion

**Fig. 6:**
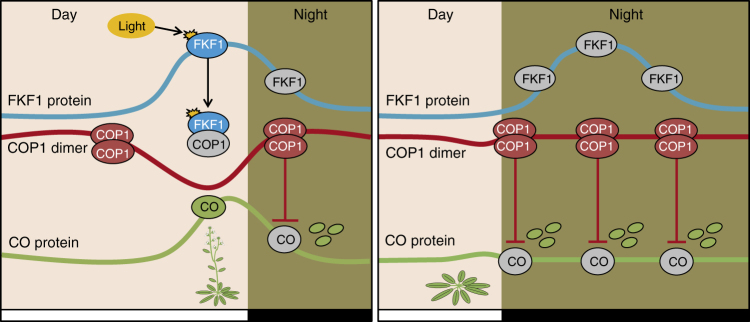
Suggested model of photoperiodic flowering through posttranslational regulation of CO by FKF1 and COP1. In LD (left panel), FKF1 protein is expressed in the afternoon and light-activated FKF1 interacts with COP1. The FKF1–COP1 interaction inhibits COP1 dimerization, resulting in stabilization of CO and early flowering. In SD (right panel), however, FKF1 protein is expressed after dusk and cannot interact with COP1 in the absence of light. Consequently, COP1 homodimers accelerate degradation of CO, resulting in late flowering. In the multi-feedforward regulatory roles of FKF1 in *Arabidopsis*, the FKF1–COP1–CO regulatory cascade could play a pivotal role in optimizing the timing of flowering under changing photoperiod conditions

For successful reproduction, most flowering plants bloom in a certain season, which they recognize mainly by sensing changes in temperature and day length. In *Arabidopsis*, CO is a key positive regulator of *FT* transcription in an LD-dependent manner, although *FT* expression is finely controlled by many regulators in other flowering pathways^2,4,6^. FKF1 and COP1 are direct positive and negative regulators, respectively, of the stability of CO^12,15^. Here, we demonstrate a direct link between FKF1 and COP1, in which FKF1 negatively regulates COP1 by the posttranslational regulation of CO. First, FKF1 genetically acts as an upstream negative regulator of COP1, as the late-flowering phenotype of the *fkf1* mutation is not present in the *cop1* background (Fig. 1a, b; Supplementary Table 1). Second, neither *FKF1* overexpression nor *fkf1* mutation alters CO abundance in the *cop1* background (Fig. 3). Third, FKF1 strongly interacts with COP1 in the presence of light (Figs. 1c–e and 4a, b). Fourth, COP1 mutant variants that are unable to dimerize are unable to function in flowering as well as photomorphogenesis (Fig. 5). Finally, the interaction between FKF1 and COP1 can inhibit COP1 homo-dimerization in a light-dependent manner (Fig. 4a, c). In summary, but, our findings show that the two important regulatory pathways for photoperiodic flowering of *Arabidopsis*, the FKF1–CO and COP1–CO pathways, that have previously been thought to act independently can act in the same pathway to regulate CO stability (Fig. 6).

*FT* is rhythmically expressed, with a peak at the end of day (around ZT16) only in LD, a few hours after the first peak of *CO* expression^9^. COP1 is a light-dependent nucleocytoplasmic partitioning protein^17,32^; however, its nuclear exclusion in darkness occurs very slowly, taking approximately 24 h^33,34^. Although it has been reported that COP1 degrades CO during nighttime, many studies, including ours (Fig. 2a, b), showed that COP1 does not function only in darkness, because CO is more stable in *cop1-4* mutants than in WT during the daytime^15,35^. COP1 levels are not altered in *FKF1* overexpressor or *fkf1-t* plants (Fig. 3). Since FKF1 accumulation is rhythmic and peaked at around ZT12–ZT16 in both LD and SD, the blue-light receptor FKF1 becomes active and interacts with COP1 in LD, but not in SD. In this scenario, it is highly possible that in the presence of COP1, CO is stabilized by FKF1 in LD enough to induce *FT* transcription; however, it is degraded rapidly and completely in SD.

Homo-dimerization of COP1 occurs through the CC domain^18,19^ and SPA1 also binds to the CC domain of COP1^36,37^. The molecular weight of a COP1 tetramer (COP1)_2_(SPA1)_2_ is approximately 440 kDa, but this tetramer is present in several multi-complexes much larger than 440 kDa in vivo^20,21^. In fact, FKF1 function (inhibiting COP1 homo-dimerization) produces different effects from that of the COP1^L170A^ mutation: FKF1 and COP1^L170A^ mutation both inhibit COP1 homo-dimerization, but FKF1 does not inhibit the COP1–SPA1 interaction (Fig. 4). Some photoreceptors, such as PHYs and CRYs, inhibit COP1 function although it is not clearly understood how they inhibit COP1 activity^21^. It has been reported that photo-excited CRY2 interacts with SPA1 and enhances the CRY2–COP1 interaction, resulting in suppression of COP1 activity and CO degradation for early flowering^27^. It is possible that FKF1 and CRY2 work together to inhibit the formation of COP1 complexes, in which CRY2 inhibits COP1–SPA1 hetero-dimerization, and FKF1 inhibits COP1 homo-dimerization; the formation of (COP1)_2_(SPA1)_2_ is totally inhibited in the late afternoon of LD. We suggest that a specific COP1 complex is destabilized by light-activated FKF1 and/or CRY2 thus stabilizing CO in a light-dependent manner. These two light-dependent regulatory mechanisms could have an important role in the regulation of COP1 complex formation for photoperiodic flowering, but this remains to be determined.

There are many possible mechanisms to explain how FKF1 inhibits COP1 activity. We provide evidence that FKF1 may negatively regulate COP1 activity by inhibiting its dimerization, but other regulatory mechanisms may exist. First, FKF1 may compete with other E2-ubiquitin conjugating enzymes (such as AtUBC9)^22^ because FKF1 binds to the RING domain of COP1 (Fig. 1d). Many E2 enzymes bind to the RING domain of RING-type E3 ligases and this interaction plays an important role in E3 activity^38^. Studies of the relationship between FKF1 and other E3 enzyme(s) are needed to understand the regulatory mechanisms of E3-ubiquitin ligases. Second, FKF1 may be involved in the nuclear exclusion of COP1. We analyzed COP1 protein accumulation in nuclear and cytoplasmic fractions from Col-0, *fkf1-t*, and *35S::FKF1*, and found that FKF1 alone is not involved in the light/dark-induced movement of COP1 (Supplementary Fig. 10). Instead, we further found that all ZTL family members (ZTL, LKP2, and FKF1) interact with COP1 (Supplementary Fig. 11), suggesting that the ZTL family may also be related to COP1 function throughout development. The functions of ZTL family members in other COP1-mediated regulatory mechanisms during growth and development remain to be determined.

Finally, experimental observations and mathematical modeling indicated that COP1 function is repressed in the light by a photoreceptor-related inhibitor termed “I”^39^. Here, we demonstrate that the blue-light receptor FKF1 is a strong candidate among the hypothesized inhibitors, because FKF1 interacts with and inhibits COP1 homo-dimerization in a light-dependent manner. It seems that FKF1-mediated regulation of both CDF1 stability and COP1 activity are required to regulate light-dependent and internal rhythm-dependent control of protein expression^40,41^. Based on these findings, we propose a new model involving an FKF1–COP1–CO cascade (Fig. 6); the inhibition of COP1 homo-dimerization by light-activated FKF1 stabilizes CO in the afternoon of LD, resulting in early flowering. In SD, however, FKF1 expression mainly occurs after dusk and an inactive form of FKF1 cannot interact with COP1, resulting in high levels of COP1 homodimers that can degrade CO completely, preventing *FT* transcription, which leads to late flowering. This FKF1–COP1–CO regulatory cascade could be another layer in previously suggested models of the FKF1–GI–CDF1–CO pathway^11,12^.

## Methods

### Plant materials and growth conditions

All *A. thaliana* plant materials including WT, mutants, and transgenic plants were in the Columbia (Col-0) ecotype; *cop1-4*^28^, *fkf1-t* (SALK_059480), *fkf1-2*^42^, *co-101*^43^ mutants, and *35S::TAP-COP1/cop1-6*^44^ plants were used in this study. We generated the *cop1-4 fkf1-t* double mutant by crossing *cop1-4* and *fkf1-t*, *35S::FKF1, 35S::Myc-FKF1*, and *35S::Myc-FKF1 #3/cop1-4* (crossing *35S::Myc-FKF1 #3* and *cop1-4*). Also, we generated COP1 variants as *35S::COP1*^*WT*^*-GFP/cop1-4, 35S::COP1*^*L105A*^*-GFP/cop1-4*, and *35S::COP1*^*L170A*^*-GFP/cop1-4* transgenic plants. To generate *COP1* variants (*COP1*^*L105A*^ and *COP1*^*L170A*^), the *COP1*^*WT*^ cDNA sequence was modified using the QuikChange II Site-Directed Mutagenesis kit with a *Pfu* turbo polymerase (Agilent, Santa Clara, USA). The mutated *COP1* cDNAs were confirmed by sequencing and introduced into the pMDC85 vector for the expression of *GFP*-tagged *COP1* constructs. Information about *COP1* mutation sites was previously reported^19^. To generate the *cop1-4 fkf1-t* double mutant, we crossed *cop1-4* with *fkf1-t*. In the F_2_ progeny, *cop1-4 fkf1-t* double mutants were selected by the Derived Cleaved Amplified Polymorphic Sequence (dCAPS) method for the *cop1-4* allele and by genotyping with the specific T-DNA confirmation primers for the *fkf1-t* allele. For identification of the *cop1-4* allele, the dCAPS-DNA fragments were amplified from genomic DNA using specific primers (5′-AGAAGGATGCGCTGAGTGGGTCAGACTAG-3′ and 5′-TGCCATTGTCCTTTTACCATTTCAGC-3′), and the PCR-amplified DNA fragment was digested with the *Spe*I restriction enzyme (Promega), which cuts the DNA of the *cop1-4* mutant. For identification of the *fkf1-t* allele, three specific primers were used (forward primer 5′-GCATGGTCGAGTAACAAGGAG-3′, reverse primer 5′-TGATGCAGAGTGTCCTGAGTG-3′, and border primer 5′-TGGTTCACGTAGTGGGCCATCG-3′). To create *35S::FKF1* transgenic plants, the *FKF1* cDNA was amplified using forward primer 5′-CACCATGGCGAGAGAACATGCGATCGGAG-3′ and reverse primer 5′-AAAGTCGACTTACAGATCCGAGTCTTGCCGGC-3′ and cloned into the pENTR/D-TOPO vector (Invitrogen). The *FKF1* cDNA was cloned into the pB7WG2 binary vector, which contains the *35S* promoter^45^. Then, the pB7WG2 binary vector carrying the *FKF1* cDNA was transformed into WT plants. The *35S::FKF1 #18* line, which possesses a single copy of the transgene, was used for the analysis. To create *35S::Myc-FKF1* transgenic plants, the full-length *FKF1* cDNA was amplified from first-strand cDNA of WT plants using gene-specific primers (5′-ATGGCGAGAGAACATGCG-3′ and 5′-TTACAGATCCGAGTCTTGCC-3′), and cloned into the pPCR8/GW/TOPO vector (Invitrogen). After the *FKF1* cDNA was confirmed by sequencing, the *FKF1* cDNA was subcloned into the pEarleyGate 203 binary vector using the LR clonase II (Invitrogen). For the *FKF1*-overexpressing transgenic plants, the *35S::Myc-FKF1* vector was transformed into WT plants. To generate *35S::Myc-FKF1 #3/cop1-4* plants, the *35S::Myc-FKF1 #3* plant was crossed with the *cop1-4* mutant. The *cop1-4* allele was selected by dCAPS and was checked in MS medium including hygromycin to select *35S::Myc-FKF1* homozygous plants in the F_3_ seeds. To generate COP1 variants (*35S::COP1*^*WT*^-*GFP/cop1-4, 35S::COP1*^*L105A*^*-GFP/cop1-4*, and *35S::COP1*^*L170A*^*-GFP/cop1-4*), the *COP1* cDNA was amplified from the first-strand cDNA of WT (Col-0) using gene-specific primers (5′-ATGGAAGAGATTTCGACGG-3′ and 5′-TCACGCAGCGAGTACCAG-3′). The PCR-amplified *COP1* was ligated into the pDONR221 vector (Invitrogen) and introduced into the pMDC85 vector for the expression of COP1-GFP by the Gateway cloning system. To generate *COP1* variants, *COP1*^*L105A*^ and *COP1*^*L170A*^, the *COP1*^*WT*^ cDNA sequence was modified using the QuikChange Site-Directed Mutagenesis Kit using a *Pfu* turbo polymerase (Agilent). The mutated *COP1* cDNAs were confirmed by sequencing and introduced into the pMDC85 vector for the expression of *GFP*-tagged *COP1* constructs. Information about *COP1* mutation sites was previously reported^18^. To generate various *COP1-GFP* transgenic plants, the binary vectors including the *35S::COP1*^*WT*^-*GFP, 35S::COP1*^*L105A*^*-GFP*, or *35S::COP1*^*L170A*^*-GFP* constructs were transformed into *cop1-4* mutant plants. Plants were grown on Murashige–Skoog (MS) phytoagar media containing 1% sucrose and 2 mM MES (pH 5.7) buffer or on soil in the growth chambers at constant 22 °C under cool white fluorescent light (100 μmol/m^2^/s) under LD (16-h light/day) or SD (8-h, 9-h, or 10-h light/day).

### Transient co-expression by co-infiltration in tobacco leaves

The cDNAs of *COP1* and *FKF1* were amplified from the first-strand cDNA of WT (Col-0), and were cloned into the pPCR8/GW/TOPO vector (Invitrogen). After each cDNA was confirmed by sequencing, it was subcloned into a binary vector using the LR clonase II (Invitrogen); *FKF1* was cloned into the pEarleyGate 201 binary vector, *COP1* into the pEarleyGate 203 binary vector, and *COP1* variants into the pMDC85 binary vector. The *35S::HA-FKF1*, *35S::Myc-COP1*, *35S::COP1*^*WT*^*-GFP*, *35S::COP1*^*L105A*^*-GFP*, and *35S::COP1*^*L170A*^*-GFP* constructs were transformed into *Agrobacterium tumefaciens* strain GV3101 by electroporation. Each transformed agrobacterium strain was cultured overnight at 30 °C in 3 mL YEP media containing antibiotics and subcultured into 50 mL YEP for overnight growth at 30 °C. Cultured cells were harvested by centrifugation and resuspended in infiltration buffer (100 mM MES, 100 mM MgCl_2_, 100 μm acetosyringone) to OD_600_ 1.0. Each transformed agrobacterium strain was mixed and infiltrated into the *N. benthamiana* leaves^30^. The infiltrated tissues were harvested at the indicated time points after 48-h incubation in LD.

### Yeast two- and three-hybrid assays

Yeast two-hybrid assays were performed using the Matchmaker GAL4 two-hybrid system (Clontech). The full and partial cDNAs of each gene were cloned into the pGADT7 and pGBKT7 vectors as prey and bait, respectively. The full and partial (RING, aa 1–104; CC, aa 121–213; WD40, aa 371–675) cDNAs of *COP1* were cloned into the pGBK vector (as baits)^23^. *FKF1* was cloned into the pGAD vector (as prey) with full and partial cDNAs (LOV, aa 1–174; LOV+F-box, aa 1–283; F-box+KELCH, aa 174–618; KELCH, aa 283–618)^11^. The clones were co-transformed into the yeast strain AH109. The pBridge vector (Clontech) was used for yeast three-hybrid assays. *COP1* or *SPA1* cDNAs were cloned into the multi-cloning site I of the pBridge vector, in which the binding domain BD-COP1 or BD-SPA1 fusion protein was expressed. Then, *FKF1* was cloned into multi-cloning site II of the pBridge vector, in which *FKF1* expression was controlled by the Met-repressible *pMET25* promoter. These vectors were co-transformed into the yeast strain AH109. Yeast transformation was performed according to the Yeast Handbook (Clontech). The colonies were used for yeast cell growth assay, and a liquid assay using chlorophenol red-β-D-galactoside (CPRG) was used to measure β-galactosidase activity.

### BiFC assays

To examine the in vivo interaction, full-length cDNAs of *COP1* and *FKF1* were cloned into the BiFC Gateway vectors^46^. Each cDNA was cloned into the pCR8/GW/TOPO vector (Invitrogen). After cDNA sequence confirmation, they were subcloned into the BiFC plasmid sets pSAT5-DEST-cEYFP(175-end)-C1 (pE3130), pSAT5(A)-DEST-cEYFP(175-end)-N1 (pE3132), pSAT4(A)-DEST-nEYFP(1-174)-N1 (pE3134), and pSAT4-DEST-nEYFP(1-174)-C1 (pE3136). Each pair of recombinant plasmids encoding nEYFP or cEYFP fusion proteins was co-bombarded into onion epidermal cell layers with a DNA particle delivery system (Biolistic PDS-1000/He, Bio-Rad), and incubated with 50 μM MG132 in MS phytoagar media for 16 h at 22 °C under continuous light, followed by image analysis using confocal laser scanning microscopy (LSM710, Carl Zeiss, Germany).

### Immunoblot and Co-IP

To detect CO protein, seedlings were grown in MS agar media under LD and SD for 10 days, and were harvested at each time point. Nuclear protein was isolated using the Plant Nuclei Isolation/Extraction Kit (Sigma) following the manufacturer’s instructions, separated by 10% SDS-PAGE, and immunoblotted with an anti-CO antibody (Santa Cruz Biotechnology, sc-33753, 1:500 dilution). To detect COP1 protein, seedlings were grown on MS agar media under LD for 10 days and harvested at specific time points. Total crude extracts were prepared using extraction buffer [100 mM Tris-HCl (pH 7.5), 150 mM NaCl, 1 mM EDTA, 0.5% NP-40, 3 mM DTT, and protease inhibitors (1 mM PMSF, 5 μg/mL leupeptin, 5 μg/mL aprotinin, 5 μg/mL pepstatin, 5 μg/mL antipain, 5 μg/mL chymostatin, 2 mM Na_2_VO_3_, 2 mM NaF and 50 μM MG132)], separated by 6% SDS-PAGE, and probed with anti-COP1 antibody. To detect FKF1 protein, seedlings were grown in MS agar media under LD and SD for 10 days. Total crude extracts were prepared using urea buffer [50 mM Tris-HCl (pH 7.5), 4 M urea, 150 mM NaCl, 0.1% NP-40, 50 μM MG132 and Protease inhibitor cocktail tablets (Roche)], separated by 12% SDS-PAGE, and immunoblotted with anti-FKF1 antibody (Santa Cruz Biotechnology, sc-12665, 1:1000 dilution). To detect HY5 protein, seedlings were grown on MS agar media under SD and continuous darkness for 5 days. Total crude extracts were prepared using urea buffer, separated by 12% SDS-PAGE, and immunoblotted with anti-HY5 antibody (Agrisera, AS12-1867, 1:500 dilution). For Co-IP assays, total protein extracts were prepared from all harvested samples using Co-IP buffer [100 mM Tris-HCl (pH 7.5), 150 mM NaCl, 1 mM EDTA, 0.5% NP-40, 3 mM DTT, and protease inhibitors (1 mM PMSF, 5 μg/mL leupeptin, 5 μg/mL aprotinin, 5 μg/mL pepstatin, 5 μg/mL antipain, 5 μg/mL chymostatin, 2 mM Na_2_VO_3_, 2 mM NaF, and 50 μM MG132)], and incubated with Protein A agarose beads (Invitrogen) to capture anti-Myc antibody (Santa Cruz Biotechnology, sc-40, 1:1000 dilution), anti-GFP antibody (Santa Cruz Biotechnology, sc-9996, 1:1000 dilution), or anti-COP1 antibody. After 2 h incubation, the beads were washed with Co-IP buffer, and eluted with SDS sample buffer at 90 °C for 1 min. The immunoprecipitated proteins were separated by 6–8% SDS-PAGE, and detected by anti-HA (for HA-FKF1, Santa Cruz Biotechnology, sc-7392, 1:1000 dilution), anti-Myc (for Myc-COP1, Santa Cruz Biotechnology, sc-40, 1:1000 dilution), anti-GFP (for COP1-GFP, Santa Cruz Biotechnology, sc-9996, 1:1000 dilution), anti-COP1, and anti-FKF1 antibodies (Santa Cruz Biotechnology, sc-12665, 1:1000 dilution) (Supplementary Fig. 12).

### Generation of anti-COP1 antibody

To generate COP1 antibody, COP1 antigen was designed to target the N-terminal region of COP1 (COP1-N; 1–305 amino-acid including the RING and CC domains). The partial cDNA encoding COP1-N was amplified from the full-length *COP1* cDNA and cloned into the pGEX-4T-3 vector to produce GST-tagged COP1 protein. The GST–COP1-N constructs were transformed into *Escherichia coli* BL21 (*DE3*) strain. Cells carrying the plasmids were grown at 37 °C to an OD_600_ of 0.8, and then the expression of GST–COP1-N protein was induced by adding 1 mM isopropyl-1-thio-β-D-galactopyranoside (IPTG) for 3 h. The proteins were predominantly in the pellet and protein was purified by elution from SDS-polyacrylamide gels using an electro-eluter (BIO-RAD). The purified GST–COP1-N protein (500 μg) was injected into rabbits every 2 weeks, and after the fourth injection, blood was gathered and the serum was separated. For affinity purification of COP1 antibody, the cDNA encoding COP1-N was cloned into the pET28a vector for 6xHis tagging, and 6xHis–COP1-N protein was induced by IPTG treatment in *E. coli* BL21 (*DE3*) strain, and purified. An anti-COP1 antibody was then purified by affinity binding with 6xHis–COP1-N recombinant protein.

### Flowering time analysis

To measure flowering time of mutants and transgenic plants in *Arabidopsis*, the plants were grown on soil at 22–24 °C in LD (16 h light: 8 h dark) and SD (9 h light: 15 h dark). When the primary inflorescence length of each plant was about 3–5 cm, we counted the number of rosette and cauline leaves and recorded the days to flowering. At least 20 plants were used for measuring flowering time, and this experiment was repeated three times with similar results.

### Quantitative real-time PCR

Total RNA was isolated from 10-day-old seedlings, using the Plant RNA Isolation Kit (Macrogene). For reverse transcription, the first-strand cDNA was prepared from 2 μg of total RNA using an M-MLV reverse-transcriptase (Promega). Relative gene expression levels were analyzed by qPCR using the Light Cycler 2.0 (Roche Diagnostics). Relative mRNA levels of each gene were normalized to the expression of *ACTIN* (AT3G18780) as a loading control. The gene-specific primers are *CO* (LP 5′-GCCTACTTGTGCATGAGCTG-3′, RP 5′-GTTTATGGCGGGAAGCAAC-3′), native *CO* (LP 5′- GGATATGGGATTGTTCCTTC-3′, RP 5′- CAAACCCATTTGCACAACAG-3′), and *ACTIN* (LP 5′- TGGGATGAACCAGAAGGATG-3′, RP 5′- AAGAATACCTCTCTTGGATTGTGC-3′).

### Data availability

The authors declare that all data supporting the findings of this study are included in the manuscript and Supplementary Information files or are available from the corresponding author upon request.

## Electronic supplementary material

Supplementary Information
