# Author Correction: The F-box protein FKF1 inhibits dimerization of COP1 in the control of photoperiodic flowering

**DOI:** 10.1038/s41467-018-02966-x

**Published:** 2018-02-02

**Authors:** Byoung-Doo Lee, Mi Ri Kim, Min-Young Kang, Joon-Yung Cha, Su-Hyun Han, Ganesh M. Nawkar, Yasuhito Sakuraba, Sang Yeol Lee, Takato Imaizumi, C. Robertson McClung, Woe-Yeon Kim, Nam-Chon Paek

**Affiliations:** 1 Department of Plant Science, Plant Genomics and Breeding Institute, Research Institute of Agriculture and Life Sciences, Seoul National University, 08826 Seoul, Republic of Korea; 2 Division of Applied Life Science (BK21Plus), PMBBRC & IALS, Gyeongsang National University, 52828 Jinju, Republic of Korea; 3 Department of Biology, University of Washington, Seattle, WA 98195-1800 USA; 4 Department of Biological Sciences, Dartmouth College, Hanover, NH 03755-3563 USA

Correction to: *Nature Communications* 10.1038/s41467-017-02476-2, published online 22 December 2017

The previously published version of this Article contained errors in Figure 5. In panel c, the second and fourth blot images were incorrectly labeled ‘α-Myc’ and should have been labelled ‘α-HA’. These errors have been corrected in both the PDF and HTML versions of the Article.

