# Supplementary material for: The F-box protein FKF1 inhibits dimerization of COP1 in the control of photoperiodic flowering
Source: Nat Commun. 2017 Dec 22;8:2259. doi: 10.1038/s41467-017-02476-2 (PMC5741637; doi:10.1038/s41467-017-02476-2)
Supplement: Supplementary file 1 — Supplementary Information [file 41467_2017_2476_MOESM1_ESM.pdf]

Supplementary Information – Lee *et al*.

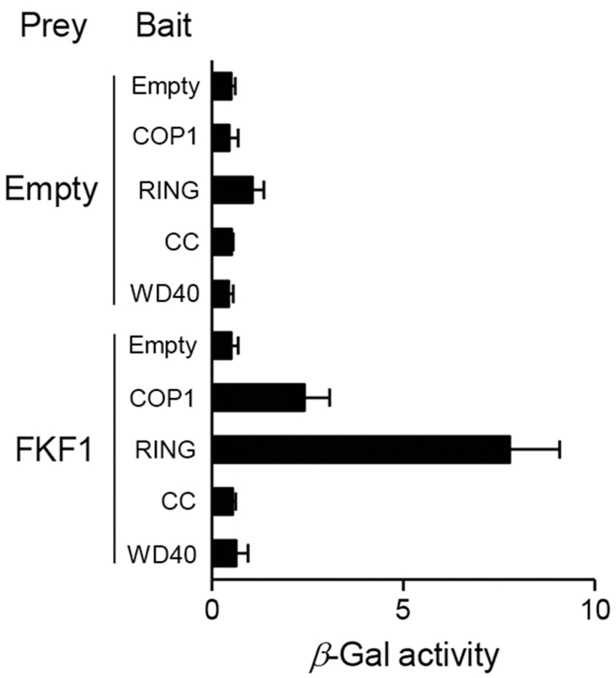

Supplementary Figure 1. FKF1 interacts with COP1 in yeast two-hybrid assays. FKF1 directly interacts with COP1. The binding activity was measured by β-galactosidase (β-Gal) activity. Data are means ± s.d. of three replicates.

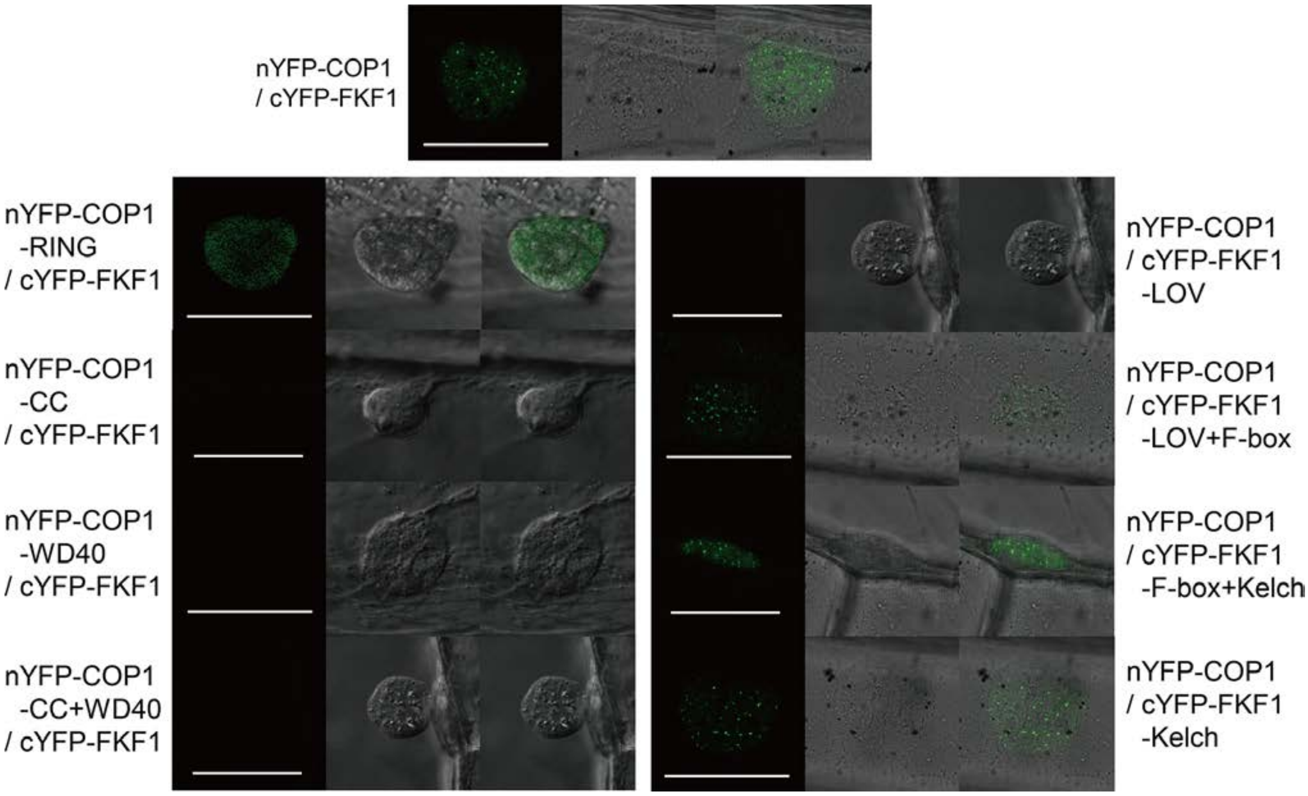

Supplementary Figure 2. BiFC assays to identify the interacting domains of FKF1 and COP1. To investigate of domain interaction between FKF1 and COP1, full-length and partial (RING, aa 1–104; CC, aa 121–213; WD40, aa 371–675; CC + WD40, aa 121–675) cDNAs of *COP1* were cloned into the pSAT5-DEST-cEYFP(175-end)-C1 (pE3130) vector, and *FKF1* was cloned into the pSAT4-DEST-nEYFP(1-174)-C1 (pE3136) vector as full and partial cDNAs (LOV, aa 1–174; LOV + F-box, aa 1–283; F-box + KELCH, aa 174–618; KELCH, aa 283–618). Each pair of recombinant plasmids encoding nEYFP or cEYFP fusion proteins was co-bombarded into onion epidermal cell layers. DIC, differential interference contrast; nY, nEYFP; cY, cEYFP; scale bars, 40 μm.

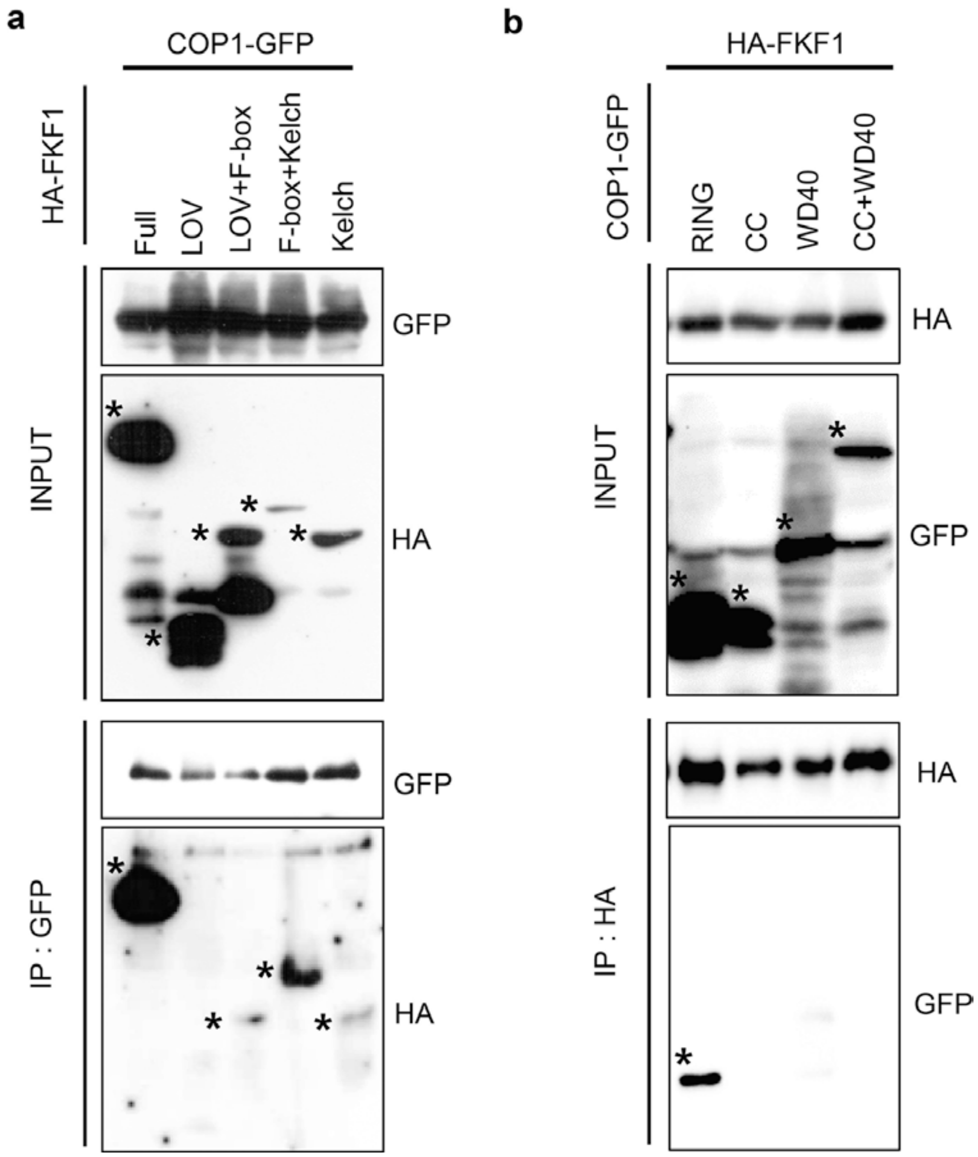

Supplementary Figure 3. Co-immunoprecipitation assays to test the interacting domains of FKF1 and COP1. (**a**) To confirm the domain interaction between FKF1 and COP1, the full and partial cDNAs (LOV, aa 1–174; LOV + F-box, aa 1–283; F-box + KELCH, aa 174–618; KELCH, aa 283–618) of *FKF1* were cloned into the pEarleyGate201 vector to produce HA-tagged fusion proteins, and the full-length cDNA of *COP1* was cloned into the pMDC85 vector to produce a GFP-tagged fusion protein. (**b**) The partial (RING, aa 1–104; CC, aa 121–213; WD40, aa 371–675; CC + WD40, aa 121–675) cDNAs of *COP1* were cloned into the pMDC85 vector to produce GFP-tagged fusion proteins, and the full-length cDNA of *FKF1* was cloned into the pEarleyGate201 vector to produce an HA-tagged fusion protein. These plasmids were transformed into *Agrobacterium tumefaciens* strain GV3101 by electroporation, and co-infiltrated into leaves of *N. benthamiana*. * indicates each protein band.

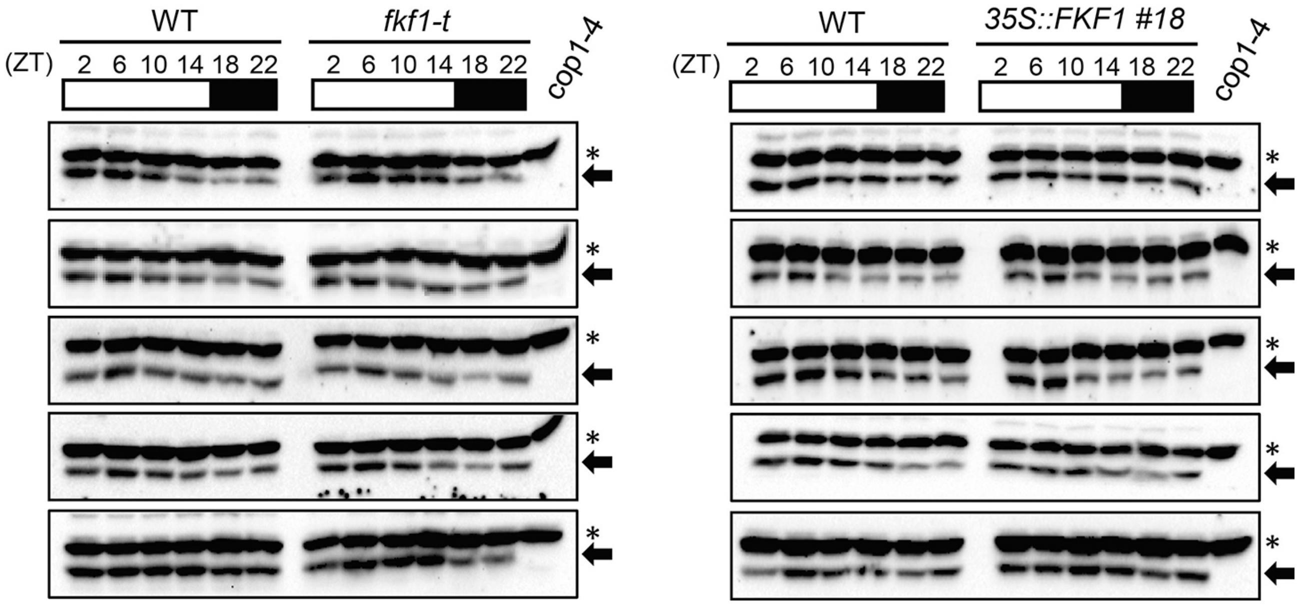

Supplementary Figure 4. The diurnal patterns of COP1 levels in *fkf1-t* and *35S::FKF1* plants. The 10-day-old seedlings grown in LD were harvested every 4 h over the course of a day. Total protein (70 µg) extracted from each sample was immunoblotted with an anti-COP1 polyclonal antibody. The intensity of each COP1 band (black arrows) was normalized to the nonspecific band (* as loading control).

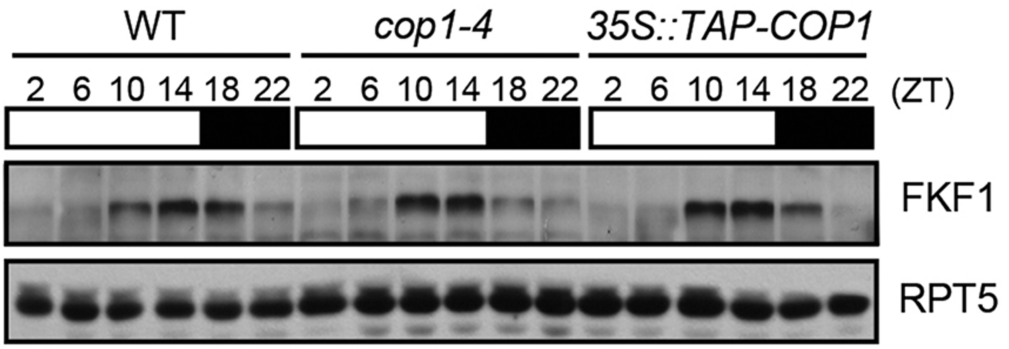

Supplementary Figure 5. The FKF1–COP1 interaction does not affect FKF1 stability. The diurnal patterns of FKF1 levels are shown in WT, *cop1-4*, and *35S::TAP-COP1* plants under LD. The 10-day-old seedlings were harvested every 4 h over the course of a day. Total protein (70 μg) extracted from each sample was immunoblotted to measure the FKF1 levels using an anti-FKF1 antibody. For a loading control, RPT5 levels were detected with an anti-RPT5 antibody.

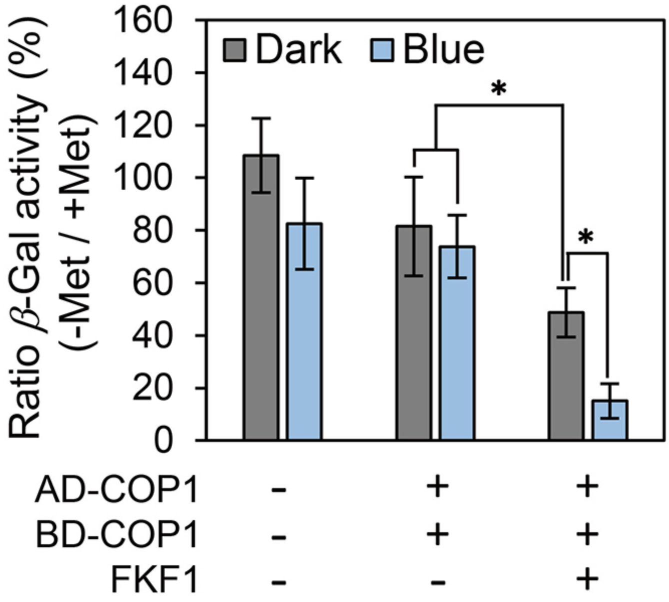

Supplementary Figure 6. FKF1 inhibits COP1 homo-dimerization more under blue light than in dark conditions. The transformed yeast cells as pGAD (empty) / pBridge (empty) vector only, pGAD-COP1 [for activation domain (AD) fused COP1] / pBridge-COP1 [for binding domain (BD) fused COP1] without FKF1, and pGAD-COP1 / pBridge-COP1 with FKF1 (in Met-repressible *pMET25* promoter), were prepared under four different conditions; 1 mM Met in dark (+Met/dark), without Met in dark (-Met/dark), 1 mM Met under blue light (+Met/blue) and without Met under blue light (-Met/blue). COP1 homodimerization was measured by activiation of the reporter gene encoding β-galactosidase and the ratios of β-galactosidase activity were calculated as [(–Met/+Met) x 100]. Data are means ± s.d from three independent yeast cells. Asterisks indicate significant difference between dark and blue light conditions (Student's *t*-test, * $P < 0.05$).

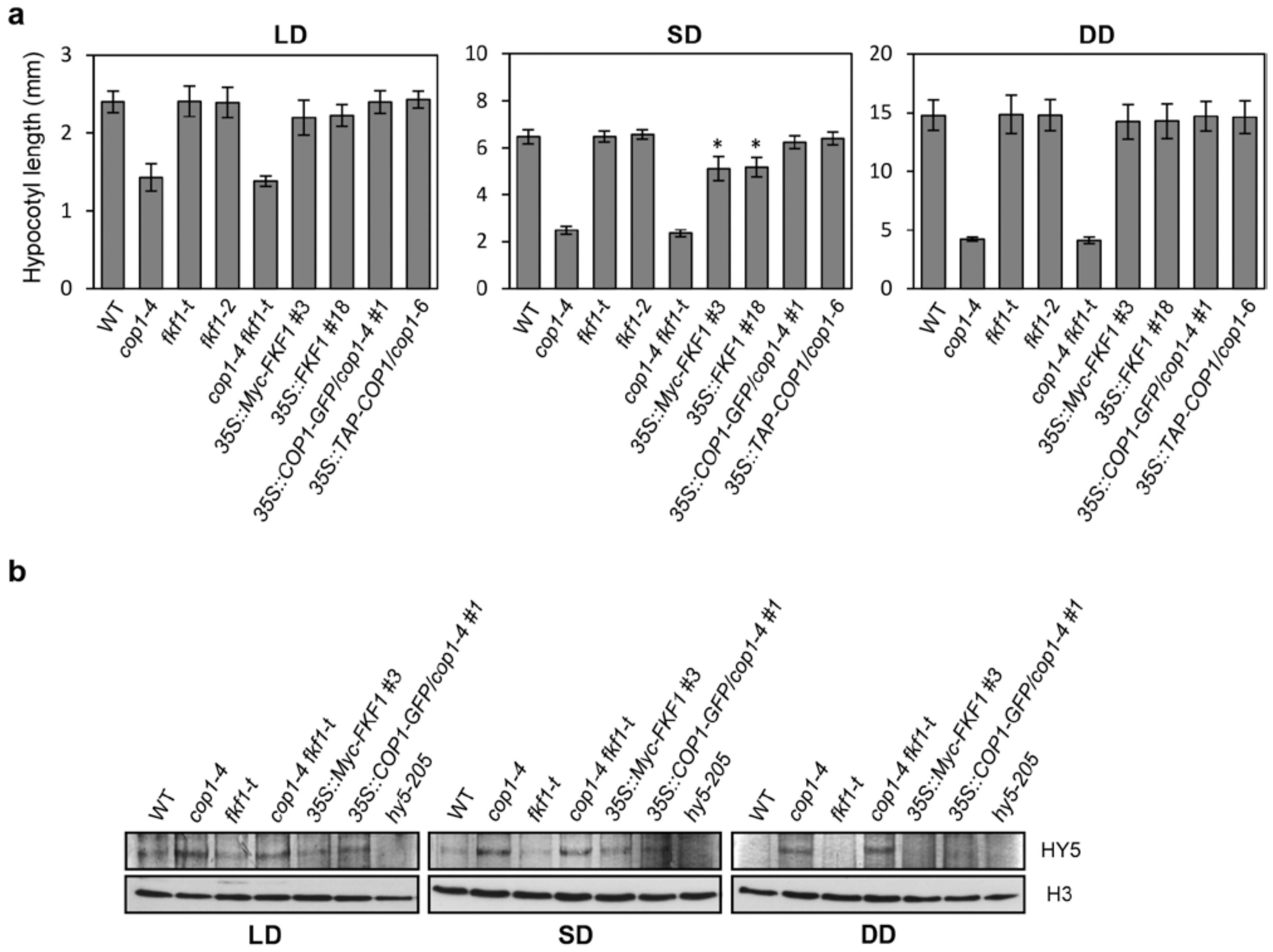

Supplementary Figure 7. FKF1 is involved in hypocotyl elongation. (**a**) Hypocotyl length in various plants. Plants were grown for 5 days in LD and SD under cool-white fluorescent light (90 μmol m^{-2} s^{-1}), or constant darkness (DD). Data are means ± standard deviations of at least 20 seedlings. (**b**) HY5 accumulation in various plants. Plants were grown for 5 days under each condition (LD/SD/DD), and harvested. Nuclear protein-enriched fractions were immunoblotted using an anti-HY5 antibody and an anti-H3 antibody for a loading control.

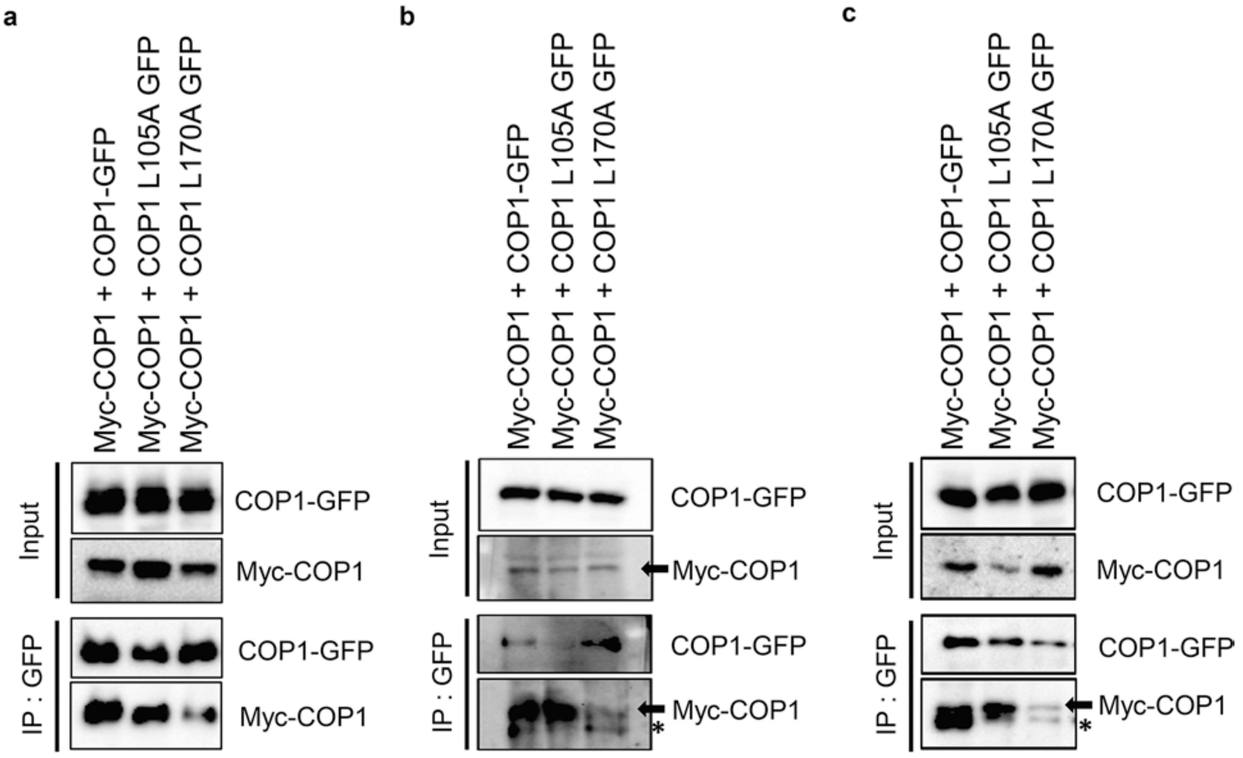

Supplementary Figure 8. COP1^{L170A} forms dimers poorly when compared with COP1^{WT} or COP1^{L105A} in *N. benthamiana*. The three replicate results showed that COP1^{L170A} was co-immunoprecipitated by COP1^{WT} but much less COP1^{L170A} was precipitated, compared with COP1^{WT} or COP1^{L105A}. (**a)** is shown in Fig. 6b, and (**b, c)** show two additional replicates of the results. The black arrows indicate Myc-COP1, and the asterisks indicate non-specific bands.

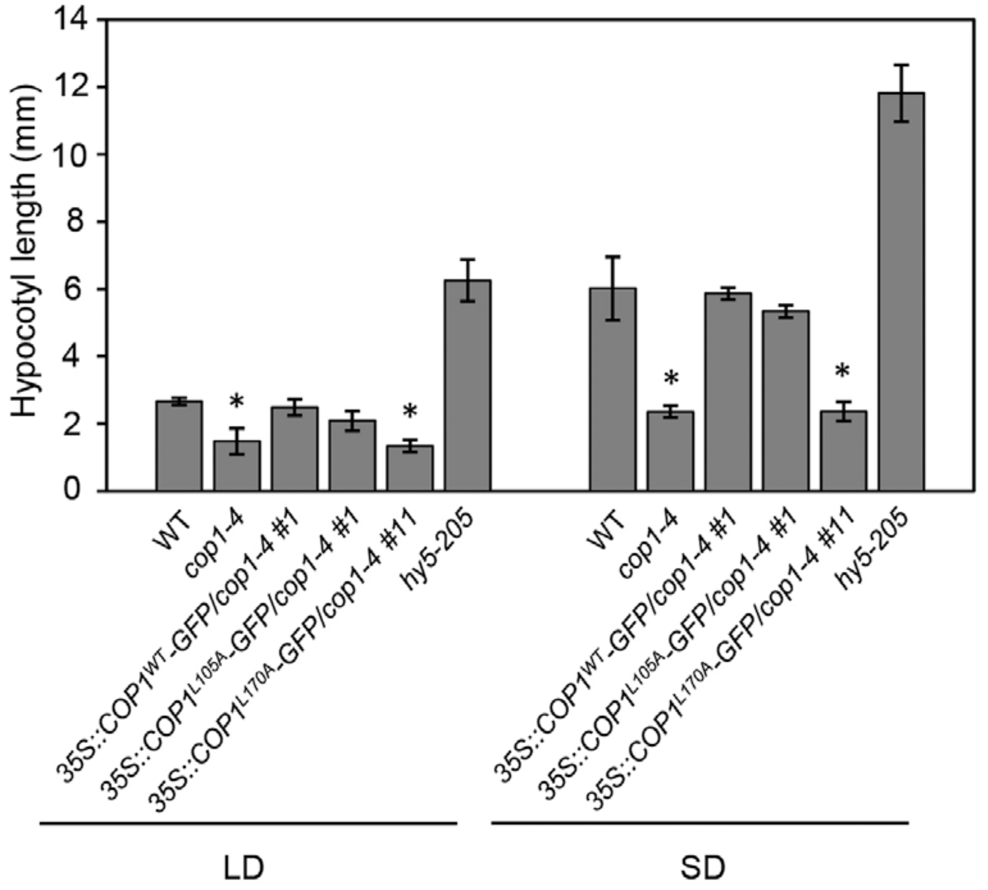

Supplementary Figure 9. Hypocotyl lengths of the transgenic plants expressing mutant forms of COP1. Plants were grown for 5 days in LD and SD under cool-white fluorescent light (90 μmol m^{-2} s^{-1}). Data are means ± standard deviations of 10 seedlings. Student's *t*-test (* *P*<0.001).

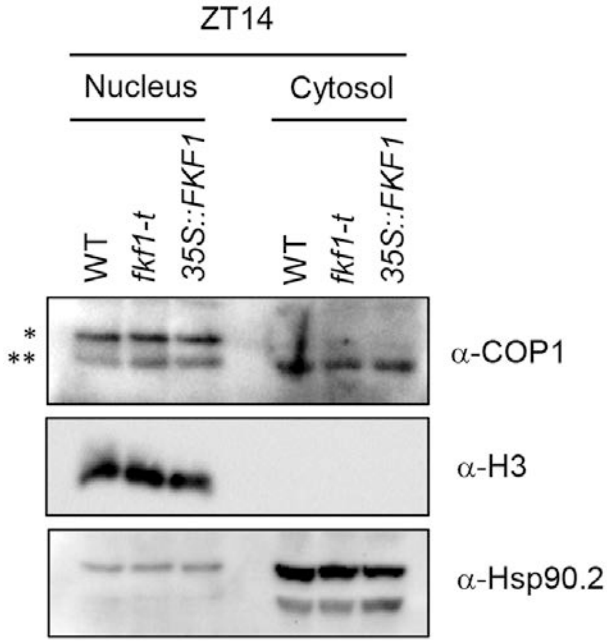

Supplementary Figure 10. FKF1 is not involved in nuclear exclusion of COP1 in LD. Ten-day-old plants grown in LD were harvested at ZT14 (2 h before dusk) in WT, *fkf1-t* and *35S::FKF1*. Nucleic and cytoplasmic protein enriched fractions were separated using the Plant Nuclei Isolation/Extraction Kit (Sigma) following the manufacturer's instructions, separated by 10% SDS-PAGE, and immunoblotted with an anti-COP1 antibody. Anti-H3 antibody and anti-Hsp90.2 antibody were used for controls (loading and fractionation). * indicates a non-specific band, ** indicates the COP1 band.

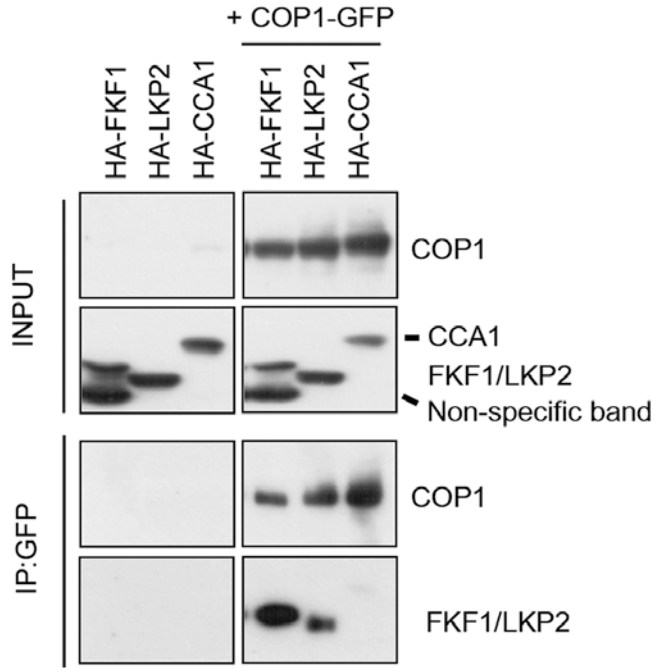

Supplementary Figure 11. COP1 interacts with ZTL family members. To investigate the interaction between COP1 and ZTL family members, we cloned COP1 into the pMDC85 binary vector (GFP tagging), and ZTL family members (ZTL/FKF1/LKP2) into the pEarleyGate 201 binary vector. These constructs were transformed into *Agrobacterium tumefaciens* strain GV3101, and co-infiltrated into the *N. benthamiana* leaves. The infiltrated tissues were harvested, immunoprecipitated by anti-COP1 antibody, and detected by anti-COP1 or anti-HA antibodies. HA-CCA1 was used for a negative control.

Supplementary Figure 12: pages 14-20.

Note: The following pages (Fig. S12) contain original images of immunoblots used in the indicated main and supplementary figures.

Fig. 1c

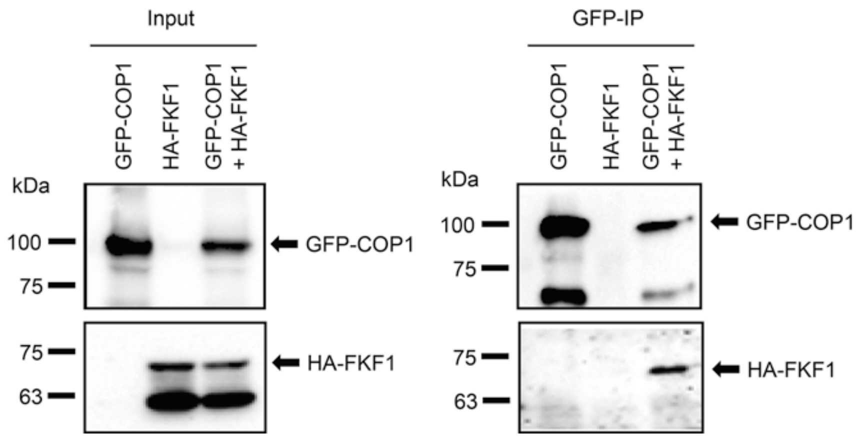

Fig. 2a

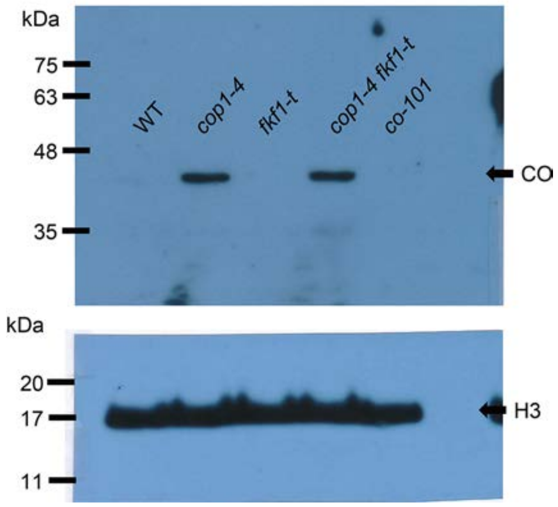

Fig. 2b

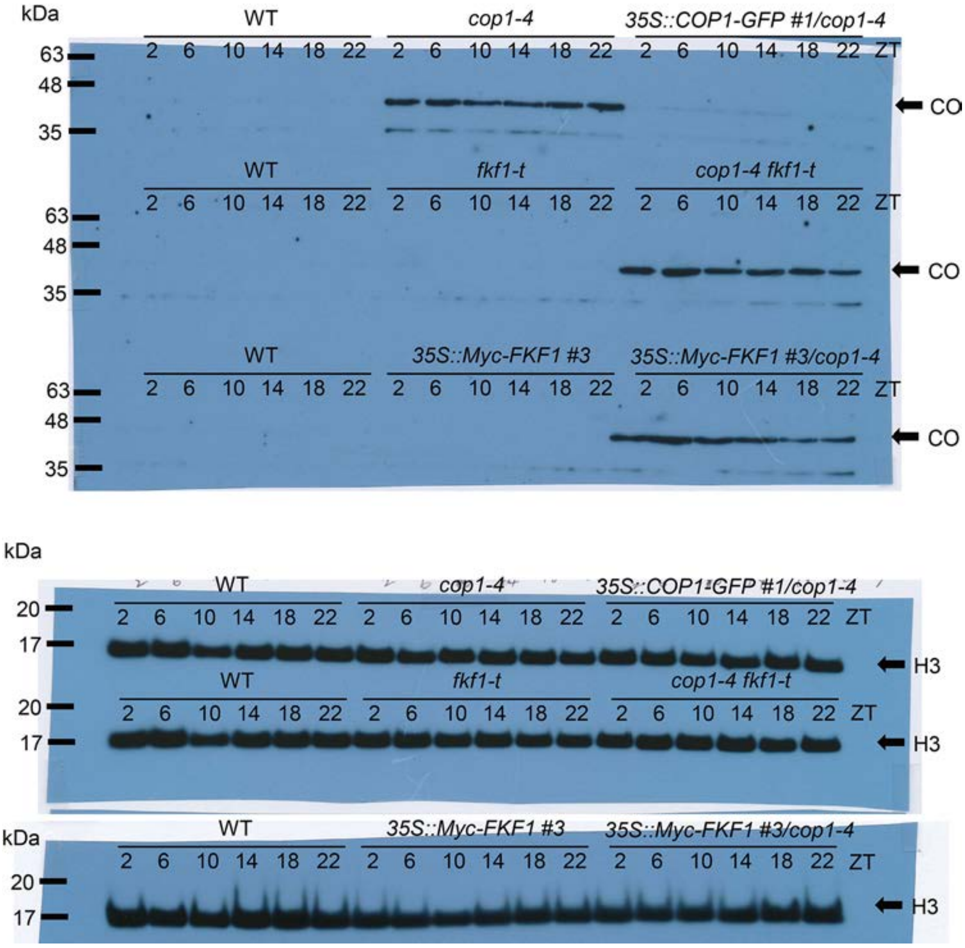
kDa
WT
cop1-4
35S::COP1-GFP #1/cop1-4
2 6 10 14 18 22 2 6 10 14 18 22 2 6 10 14 18 22 ZT
63
48
35
CO
WT
fkf1-t
cop1-4 fkf1-t
2 6 10 14 18 22 2 6 10 14 18 22 2 6 10 14 18 22 ZT
63
48
35
CO
WT
35S::Myc-FKF1 #3
35S::Myc-FKF1 #3/cop1-4
2 6 10 14 18 22 2 6 10 14 18 22 2 6 10 14 18 22 ZT
63
48
35
CO
kDa
WT
cop1-4
35S::COP1-GFP #1/cop1-4
2 6 10 14 18 22 2 6 10 14 18 22 2 6 10 14 18 22 ZT
20
17
H3
WT
fkf1-t
cop1-4 fkf1-t
2 6 10 14 18 22 2 6 10 14 18 22 2 6 10 14 18 22 ZT
20
17
H3
kDa
WT
35S::Myc-FKF1 #3
35S::Myc-FKF1 #3/cop1-4
2 6 10 14 18 22 2 6 10 14 18 22 2 6 10 14 18 22 ZT
20
17
H3

Fig. 3

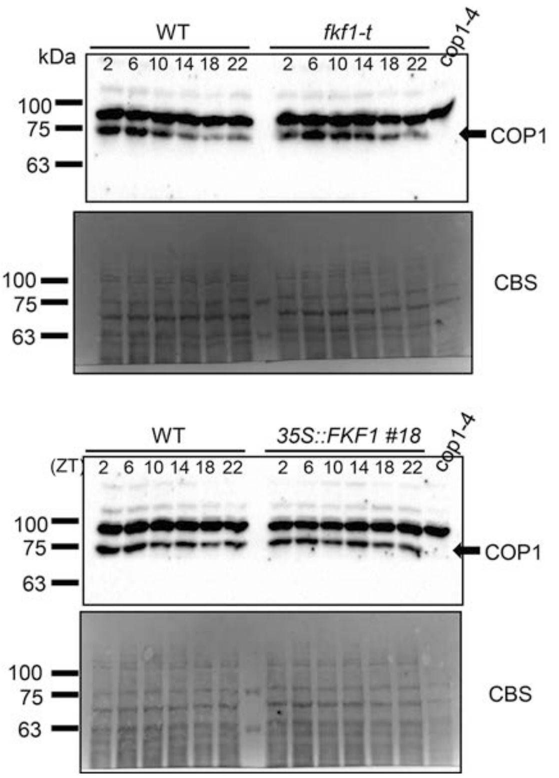
WT
fkf1-t
cop1-4
kDa
2 6 10 14 18 22
2 6 10 14 18 22
100
75
63
COP1
CBS
WT
35S::FKF1 #18
cop1-4
(ZT)
2 6 10 14 18 22
2 6 10 14 18 22
100
75
63
COP1
100
75
63
CBS

Fig. 4a

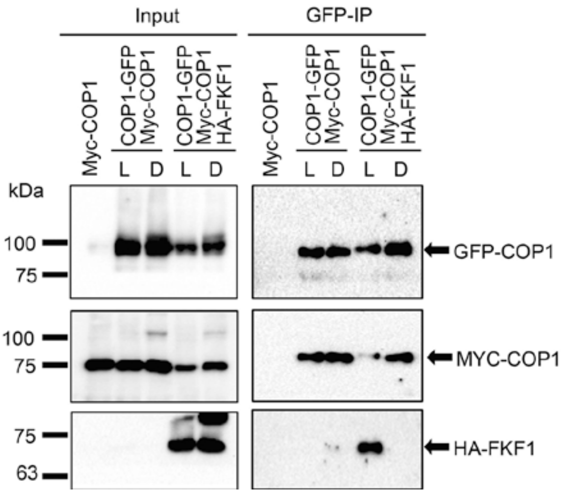

Fig. 4b

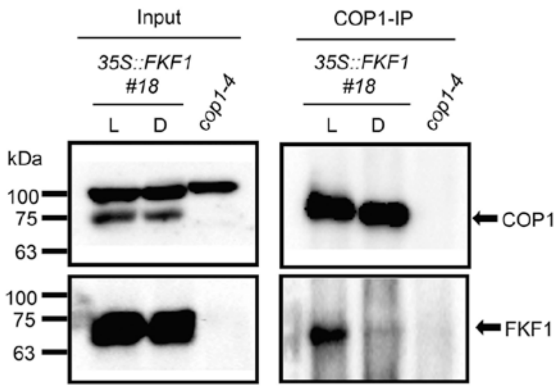

Fig. 5b

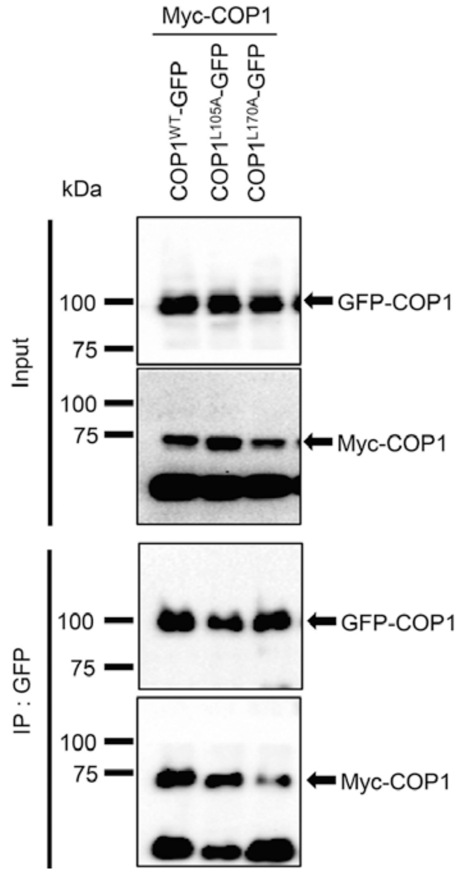

Fig. 5c

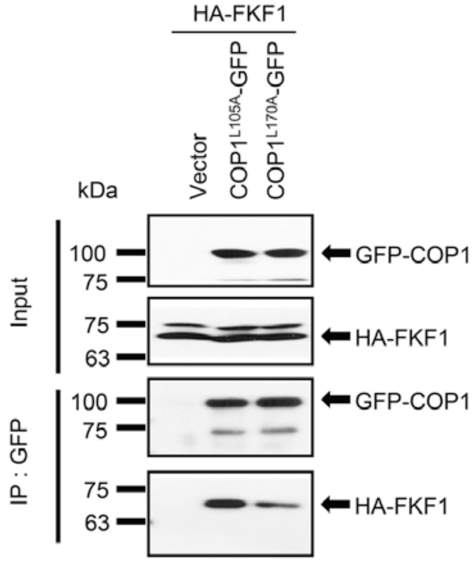

Fig. 5f

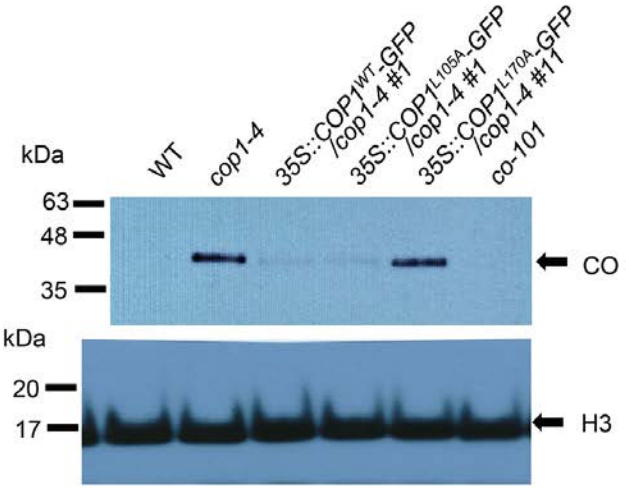

kDa
WT
cop1-4
35S::COP1^{WT}-GFP /cop1-4 #1
35S::COP1^{L105A}-GFP /cop1-4 #1
35S::COP1^{L170A}-GFP /cop1-4 #11
co-101
63
48
35
CO
kDa
20
17
H3

Fig. 5i

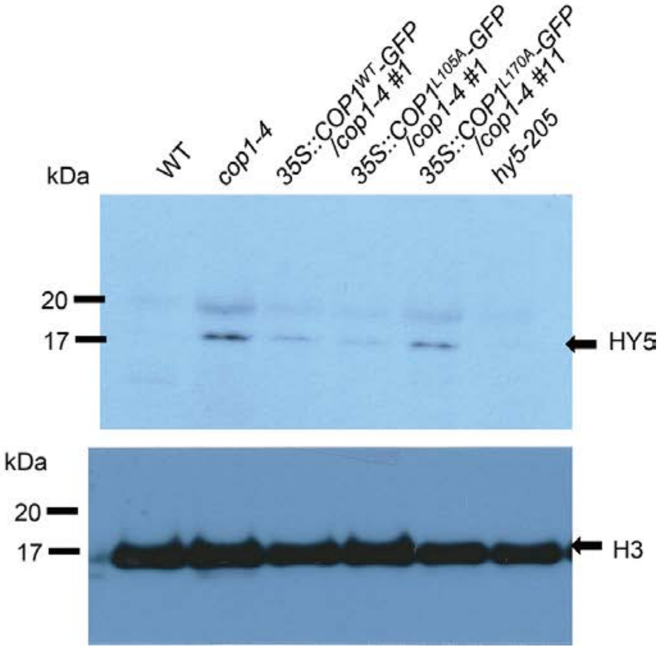

kDa
WT
cop1-4
35S::COP1^{WT}-GFP /cop1-4 #1
35S::COP1^{L105A}-GFP /cop1-4 #1
35S::COP1^{L170A}-GFP /cop1-4 #11
hy5-205
20
17
HY5
kDa
20
17
H3

Fig. S3a

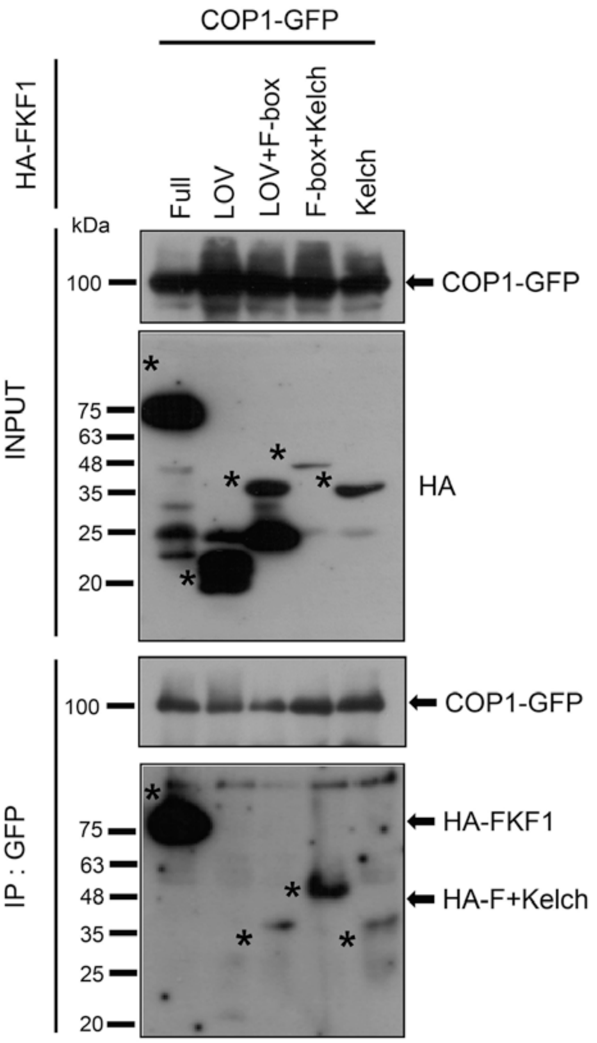

Fig. S3b

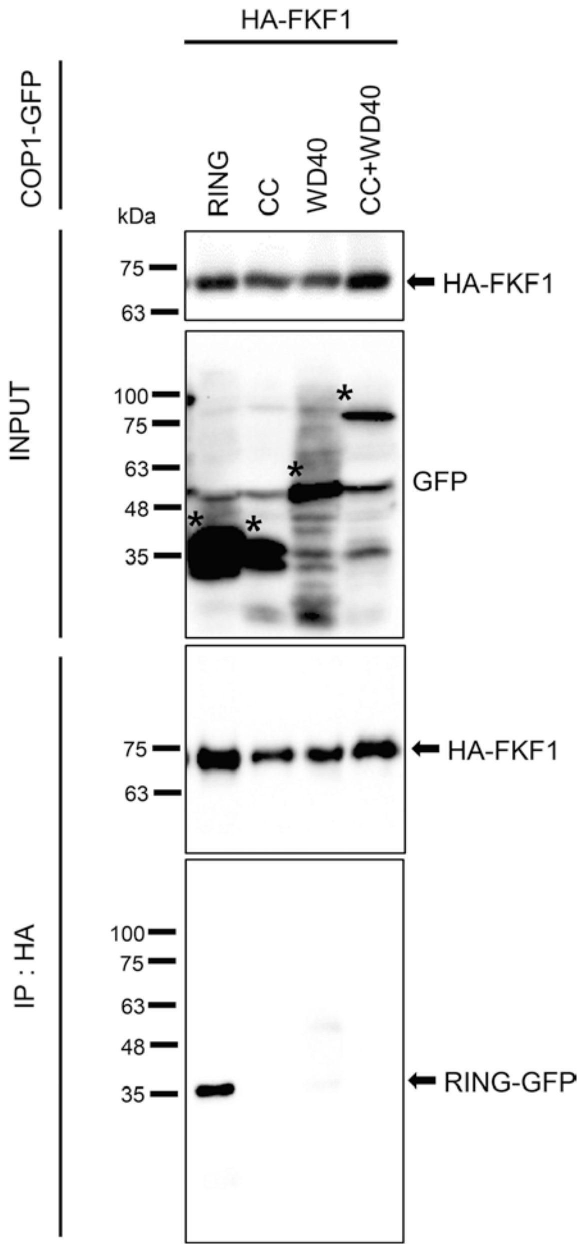

Fig. S4

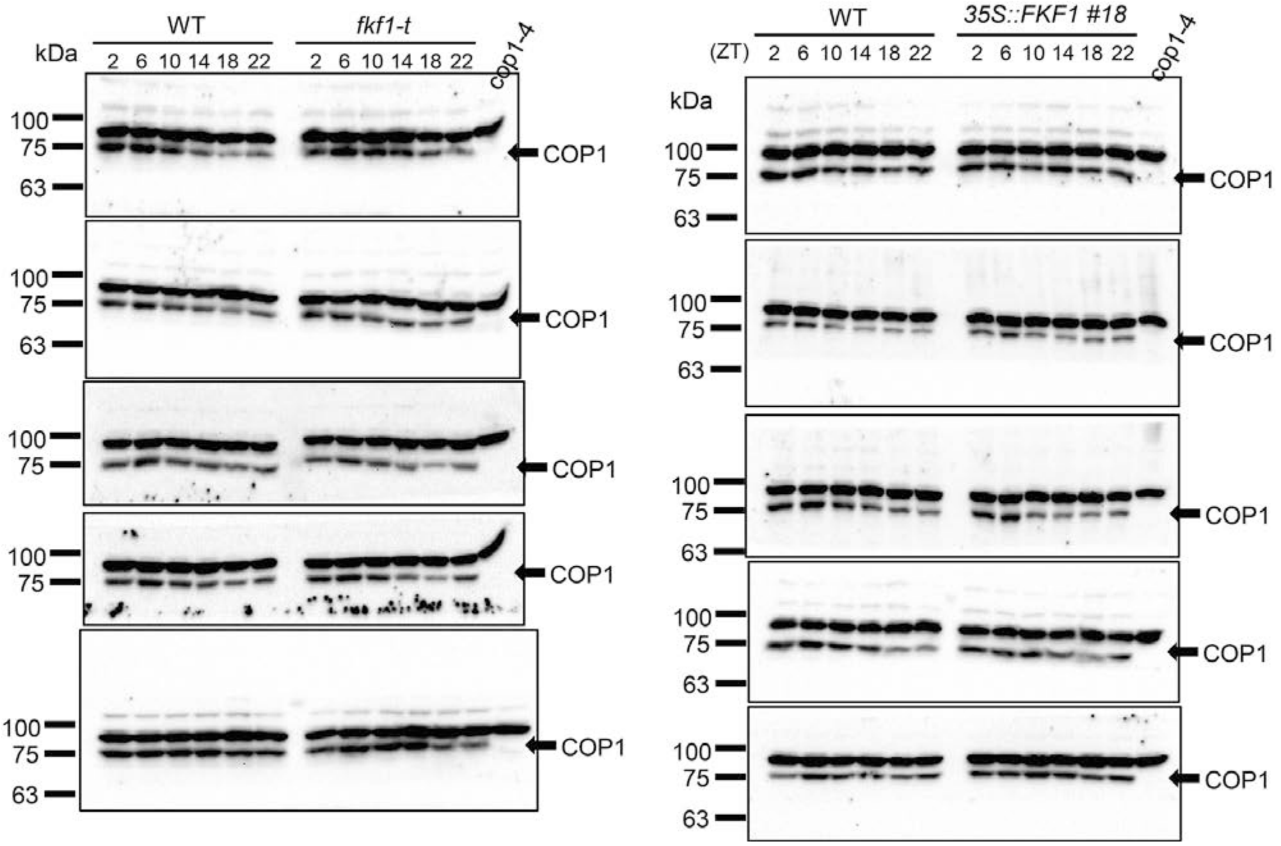
WT
fkf1-t
kDa
2 6 10 14 18 22
cop1-4
100
75
63
COP1
35S::FKF1 #18
(ZT)

Fig. S5

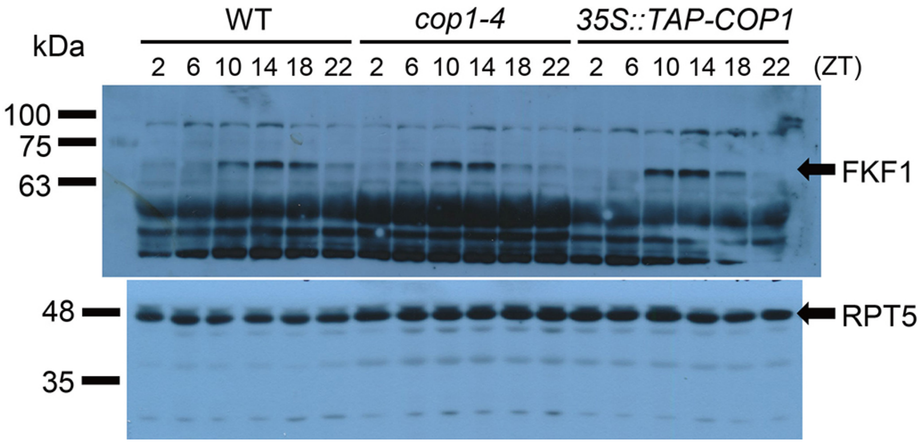
WT
cop1-4
35S::TAP-COP1
kDa
2 6 10 14 18 22
(ZT)
100
75
63
FKF1
48
35
RPT5

Fig. S7b

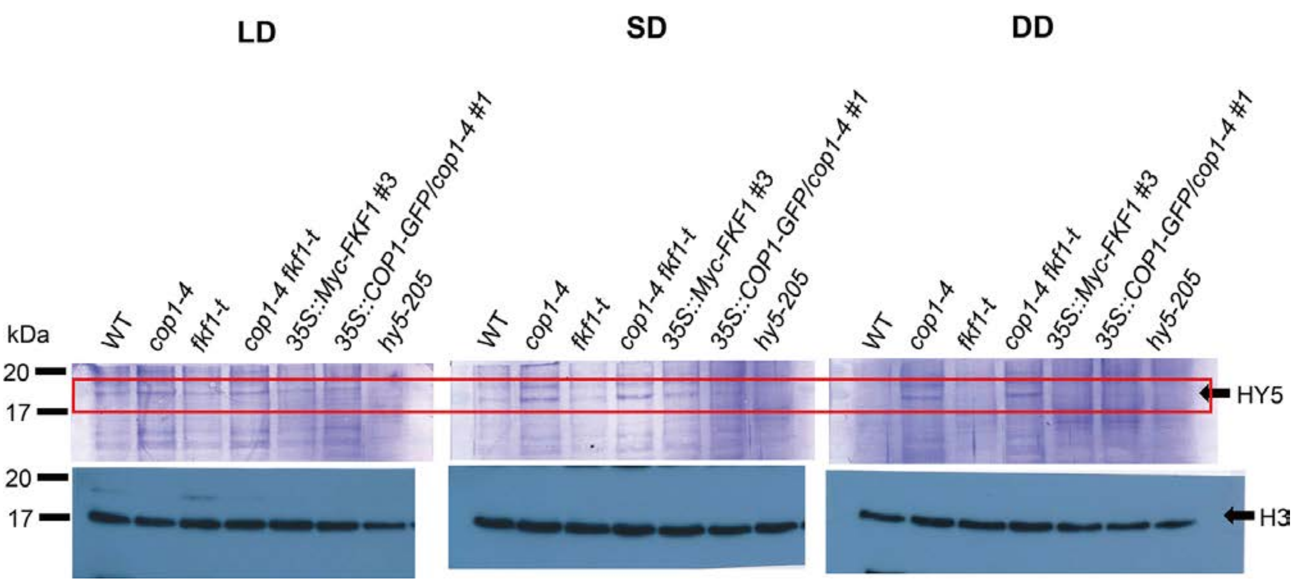

Fig. S8a

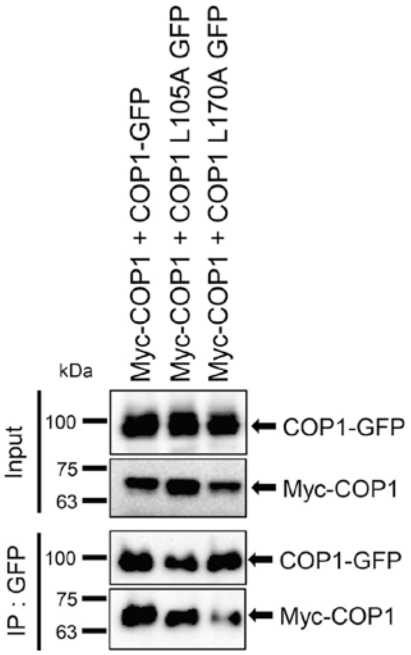

Fig. S8b

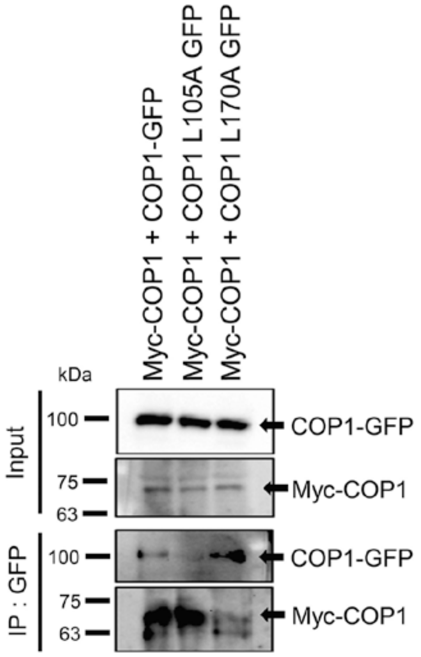

Fig. S8c

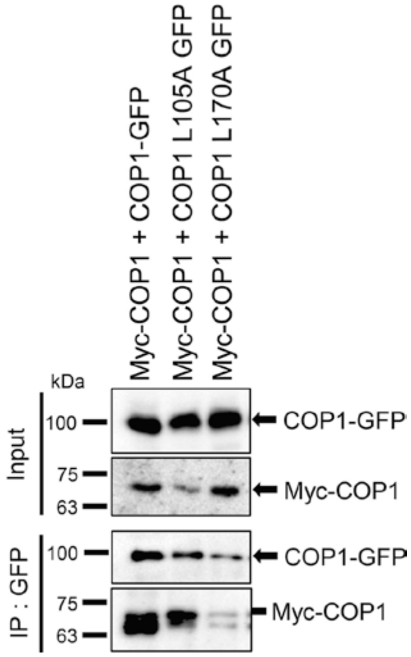

Fig. S10

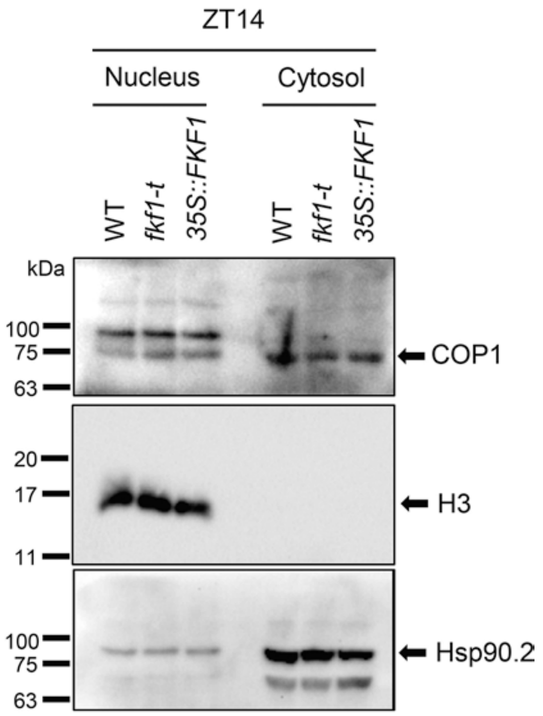

Fig. S11

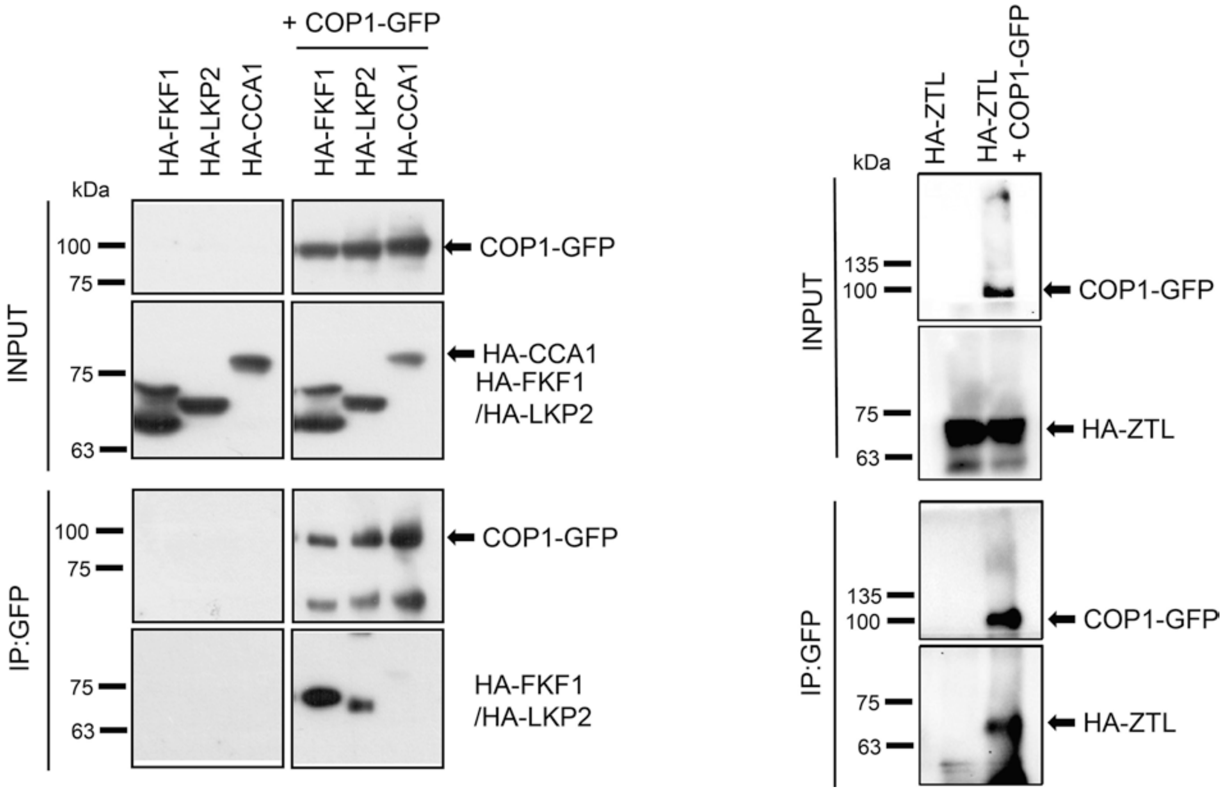

Supplementary Figure 12. Uncropped images of immunoblots. Blots shown as cropped images in various main and supplementary figures are shown as uncropped images here.

Supplementary Table 1. Flowering time of different genotypes in LD and SD.

	No. of rosette leaves		No. of total leaves		days to bolting	
Genotype	LD	SD	LD	SD	LD	SD
WT	12.0 ± 1.0	50.1 ± 2.3	14.5 ± 1.1	59.6 ± 2.4	30.5 ± 1.7	66.1 ± 2.3
cop1-4	11.3 ± 0.9	11.4 ± 0.7	13.1 ± 1.0	13.2 ± 1.1	30.0 ± 3.0	40.2 ± 3.5
fkf1-t	36.5 ± 4.3	44.2 ± 3.7	42.6 ± 5.0	52.9 ± 3.9	44.0 ± 2.0	67.1 ± 3.2
fkf1-2	37.5 ± 4.5	47.9 ± 5.1	44.4 ± 4.7	57.1 ± 6.3	45.8 ± 2.9	61.2 ± 3.4
cop1-4 fkf1-t	10.9 ± 1.2	12.1 ± 1.0	12.8 ± 1.3	14.2 ± 1.4	31.7 ± 1.3	44.3 ± 1.0
35S::Myc-FKF1 #3	9.2 ± 0.9	40.8 ± 3.1	11.7 ± 1.1	48.0 ± 3.6	27.3 ± 2.3	63.3 ± 3.2
35S::FKF1 #18	9.3 ± 0.5	40.3 ± 7.1	11.5 ± 0.6	50.5 ± 7.1	26.3 ± 1.2	61.6 ± 5.4
35S::Myc-FKF1 #3 / cop1-4	9.3 ± 1.1	10.8 ± 0.9	10.9 ± 1.3	12.4 ± 1.2	27.6 ± 1.6	44.7 ± 1.5

Plants were grown at 22–24°C in LD and SD. Flowering time of each genotype was measured as the number of rosette leaves and total leaves, and days at bolting. Data are means ± standard deviations of at least 15 plants.

Supplementary Table 2. Hypocotyl lengths of different genotypes under different light conditions.

	Hypocotyl length (mm)		
Genotype	LD	SD	DD
hy5-205	6.22 ± 0.25	10.96 ± 1.09	15.06 ± 1.68
WT	2.40 ± 0.14	6.47 ± 0.30	14.79 ± 1.30
cop1-4	1.43 ± 0.17	2.49 ± 0.18	4.22 ± 0.17
fkf1-t	2.41 ± 0.20	6.48 ± 0.23	14.86 ± 1.64
fkf1-2	2.39 ± 0.20	6.57 ± 0.20	14.80 ± 1.33
cop1-4 fkf1-t	1.38 ± 0.07	2.36 ± 0.15	4.10 ± 0.28
35S::Myc-FKF1 #3	2.20 ± 0.22	5.11 ± 0.51	14.22 ± 1.48
35S::FKF1 #18	2.23 ± 0.14	5.17 ± 0.41	14.29 ± 1.48
35S::COP1-GFP/cop1-4 #1	2.40 ± 0.14	6.24 ± 0.27	14.71 ± 1.27
35S::TAP-COP1/cop1-6	2.43 ± 0.11	6.40 ± 0.27	14.63 ± 1.39

Plants were grown for 5 days in LD (16-h light: 8-h dark) and SD (8-h light: 16-h dark) under cool-white fluorescent light (90 μmol m^{-2} s^{-1}), or constant darkness (DD). Data are means ± standard deviations of at least 20 seedlings.

Supplementary Table 3. Flowering times of the transgenic plants expressing mutant forms of COP1 in LD and SD.

	No. of rosette leaves		No. of total leaves		days to bolting	
Genotype	LD	SD	LD	SD	LD	SD
WT	12.8 ± 0.4	54.5 ± 3.0	16.0 ± 1.2	63.0 ± 3.1	26.2 ± 1.3	58.2 ± 5.7
cop1-4	11.7 ± 0.8	15.3 ± 0.8	13.3 ± 1.0	18.4 ± 1.3	27.3 ± 0.8	36.7 ± 0.8
35S::COP1^{WT}-GFP/cop1-4 #1	12.8 ± 0.8	65.3 ± 4.2	16.8 ± 1.5	73.7 ± 4.5	25.0 ± 1.7	66.0 ± 2.5
35S::COP1^{L105A}-GFP/cop1-4 #1	12.8 ± 0.8	65.0 ± 6.3	15.4 ± 1.1	73.2 ± 5.8	25.2 ± 1.1	61.8 ± 2.6
35S::COP1^{L170A}-GFP/cop1-4 #11	12.9 ± 1.0	17.3 ± 1.4	15.4 ± 1.6	20.1 ± 1.6	25.5 ± 1.0	35.6 ± 0.7

Plants were grown at 22–24°C in LD and SD. Flowering time of each genotype was measured as the number of rosette leaves and total leaves, and days at bolting. Data are means ± standard deviations of at least 20 plants.

Supplementary Table 4. Hypocotyl lengths of *COP1-GFP* transgenic lines in different light conditions.

	Hypocotyl length (mm)		
Genotype	LD	SD	DD
hy5-205	6.3 ± 0.6	11.8 ± 0.8	17.1 ± 0.6
WT	2.7 ± 0.1	6.0 ± 0.9	17.1 ± 1.8
cop1-4	1.5 ± 0.4	2.4 ± 0.2	5.3 ± 0.7
35S::COP1^{WT}-GFP/cop1-4 #1	2.5 ± 0.2	5.9 ± 0.2	16.8 ± 0.6
35S::COP1^{L105A}-GFP/cop1-4 #1	2.1 ± 0.3	5.3 ± 0.2	16.6 ± 1.3
35S::COP1^{L170A}-GFP/cop1-4 #11	1.3 ± 0.2	2.4 ± 0.3	7.1 ± 1.1

Plants were grown for 5 days in LD (16-h light: 8-h dark) and SD (8-h light: 16-h dark) under cool-white fluorescent light (90 µmol m^{-2} s^{-1}), or constant darkness (DD). Data are means ± standard deviations of at least 10 seedlings.
